# Supplementary material for: Root stem cell homeostasis in Arabidopsis involves cell-type specific transcription factor complexes
Source: EMBO Rep. 2025 Mar 19;26(9):2323–46. doi: 10.1038/s44319-025-00422-8 (PMC12069552; doi:10.1038/s44319-025-00422-8)
Supplement: Supplementary file 1 — Appendix [file 44319_2025_422_MOESM1_ESM.pdf]

Appendix for 'Root stem cell homeostasis in *Arabidopsis* involves cell type specific transcription factor complexes'

Table of Contents

Appendix Figure S1: *pBRAVO:BRAVO-mV* rescues the *bravo-2* root SCN phenotype.. 3
Appendix Figure S2: Quantification of root length measurements of *Arabidopsis thaliana* seedlings 10 DAG. 5
Appendix Figure S3: Interaction and affinity of PLT3 and WOX5 is altered upon loss of PLT3 PrDs. 6
Appendix Figure S4: Association and dissociation parameters predicted for the heterodimers and trimeric complex modelled. 8
Appendix Figure S5: Robustness of the protein complex cell signatures. 9
Appendix Figure S6:Controls for *in silico* prediction of protein complex signatures in the WT root SCN. 10
Appendix Figure S7: Trimeric complex formation of WOX5, BRAVO and PLT3 is only mildly affected upon loss of PLT3 PrDs. 12
Appendix Figure S8: Control simulations to compare fast and slow dynamics of all parameter settings describing protein complex formation. 14

Appendix Table S1: List of primers used for cloning. Italic bases represent overhangs and *Bsa*I recognition sites necessary for GreenGate cloning. 16
Appendix Table S2: List of primers used for genotyping...................................... 16
Appendix Table S3: List of entry vectors used for GreenGate cloning. 17
Appendix Table S4: List of expression vectors for stable transformation of *A. thaliana* or transient transformation of *N. benthamiana* generated in this study. 18
Appendix Table S5: List of Arabidopsis mutants and transgenic lines used in this study. 19
Appendix Table S6: Fluorescence intensities of *pPLT3:PLT3-mV, pBRAVO:BRAVO-mV* and *pWOX5:WOX5-mV* translational reporter in different cell types corresponding to Fig 1, Fig 5 and Fig 7. 20

Appendix Table S7: Average number of QC divisions and CSC layers per root related to Appendix Fig S1. 22
Appendix Table S8: Ratio of periclinal cell division planes in the QC related to Appendix Fig S1. 22
Appendix Table S9: Average number of QC divisions and CSC layers per root related to Fig 2 and Fig EV1. 23
Appendix Table S10: Ratio of periclinal cell division planes in the QC related to Fig EV1. 23
Appendix Table S11: Root length of different *Arabidopsis thaliana* genotypes at 10 DAG in mm related to Appendix Fig S2. 23
Appendix Table S12: Measured FRET efficiency and Binding values related to Fig 3, Fig 5 and Fig 7. 24
Appendix Table S13: FRET efficiencies and Binding related to Fig 4 and Fig 5. .. 26
Appendix Table S14: FRET efficiencies and Binding related to Fig EV2 and Fig 7. 27
Appendix Table S15: Additional FRET efficiencies and Binding used for Fig 5, Fig 7 and Appendix Fig S3. 28
Appendix Table S16: FRET efficiencies and Binding related to Fig 6 and Fig 7. .. 30
Appendix Table S17: FRET efficiency and Binding values corresponding to Fig EV4. 32
Appendix Table S18: FRET efficiency and Binding values corresponding to Fig EV4. 31
Appendix Table S19: Ratio of periclinal cell divisions in the QC related to Fig 7. . 32
Appendix Table S20: FRET efficiencies and Binding related to Appendix Fig S7. 33

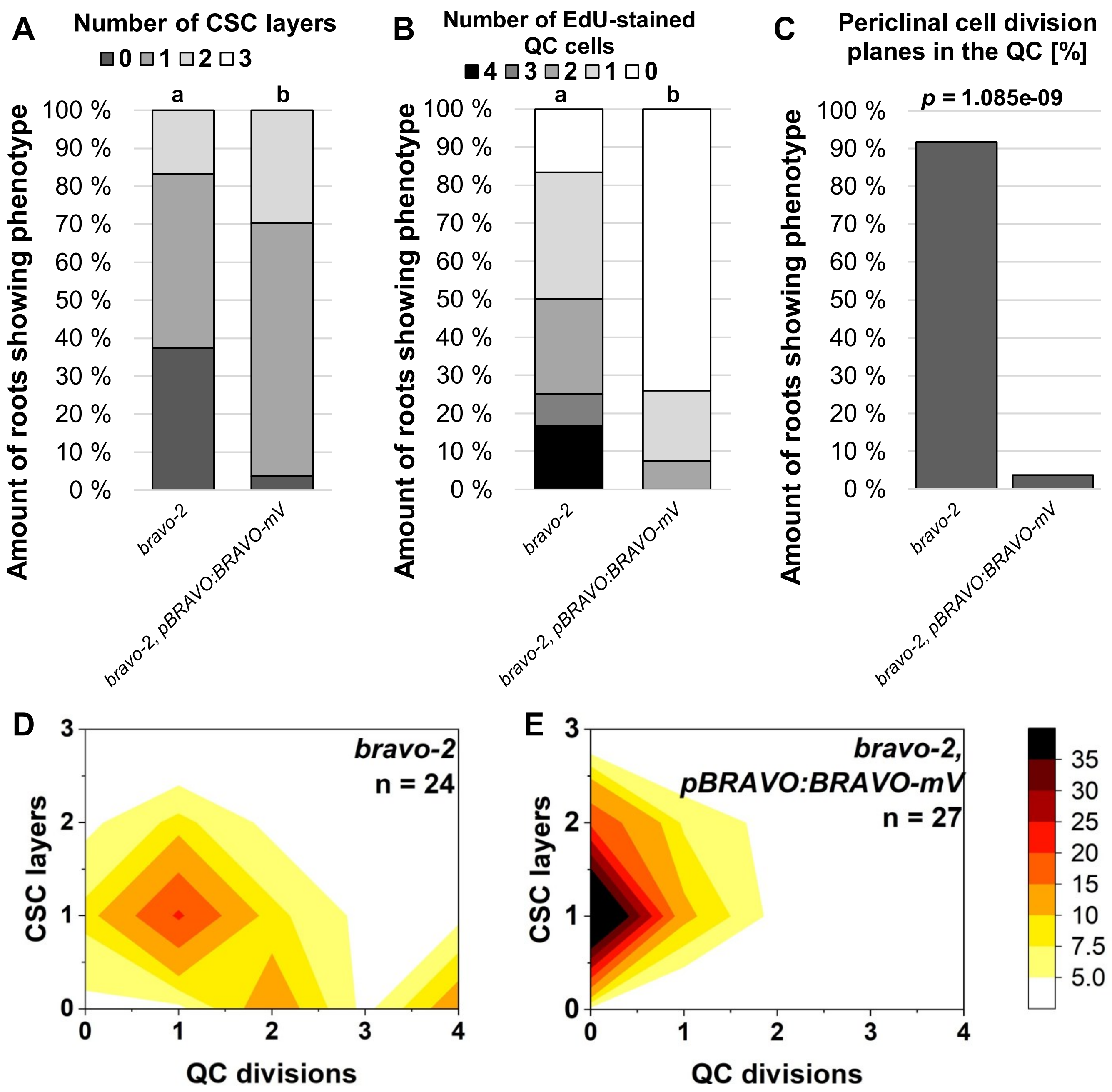

Appendix Figure S1. *pBRAVO:BRAVO-mV* rescues the *bravo-2* root SCN phenotype.

A Quantification of SCN staining displaying 0, 1, 2 or 3 CSC layers.

B Quantification of QC division frequency from 0 to 4 or more.

C Quantification of additional periclinal cell division planes in the QC.

D, E 2D histograms combining number of CSC layers and QC division frequency from the SCN staining.

Data information: *bravo-2*: n = 24 (biological replicates); *bravo-2, pBRAVO:BRAVO-mV*: n = 27 (biological replicates), resulting from two technical replicates. **A, B** Statistical groups for QC division frequency and CSC layer number were assigned after a non-parametric Kruskal-Wallis with *post-hoc* Dunn's test ($\alpha = 0.05$, *p*-values were adjusted after Benjamini and Hochberg). **C** Correlations between periclinal QC divisions and genotype were tested with Pearson's Chi-squared test.

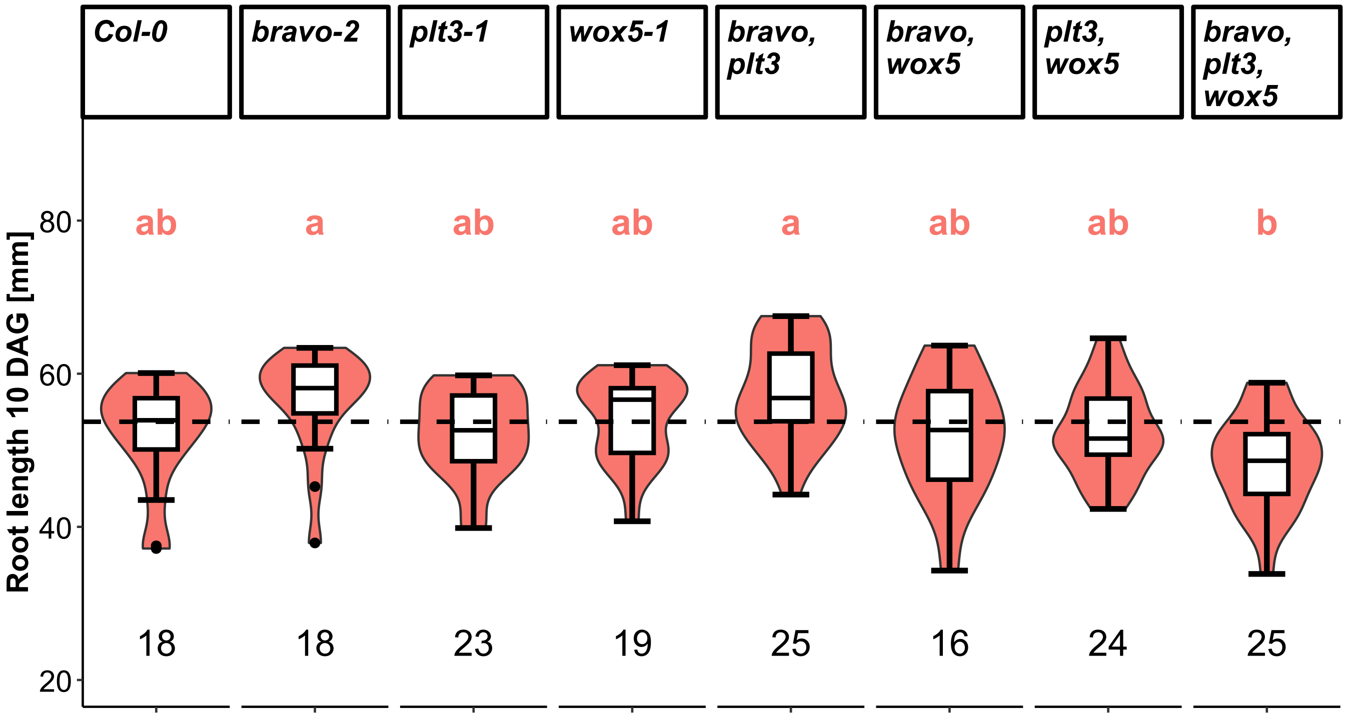

Appendix Figure S2. Quantification of root length measurements of *Arabidopsis thaliana* seedlings 10 DAG.

Statistical groups were assigned after a non-parametric Kruskal Wallis with *post-hoc* Dunn's test (α = 0.05, *p*-values were adjusted after Benjamini and Hochberg). The numbers of analysed roots (biological replicates) are indicated below each box/violin plot and results from three technical replicates. DAG = days after germination.

Data Information: Box = middle 50 % of data (= interquartile range (IQR)); whiskers = from IQR to min/max values, but at most 1.5 * IQR; line within box = median; data beyond the end of whiskers are ‚outliers' and are plotted independently. In addition, a jitter plot was used to visualize all data points individually.

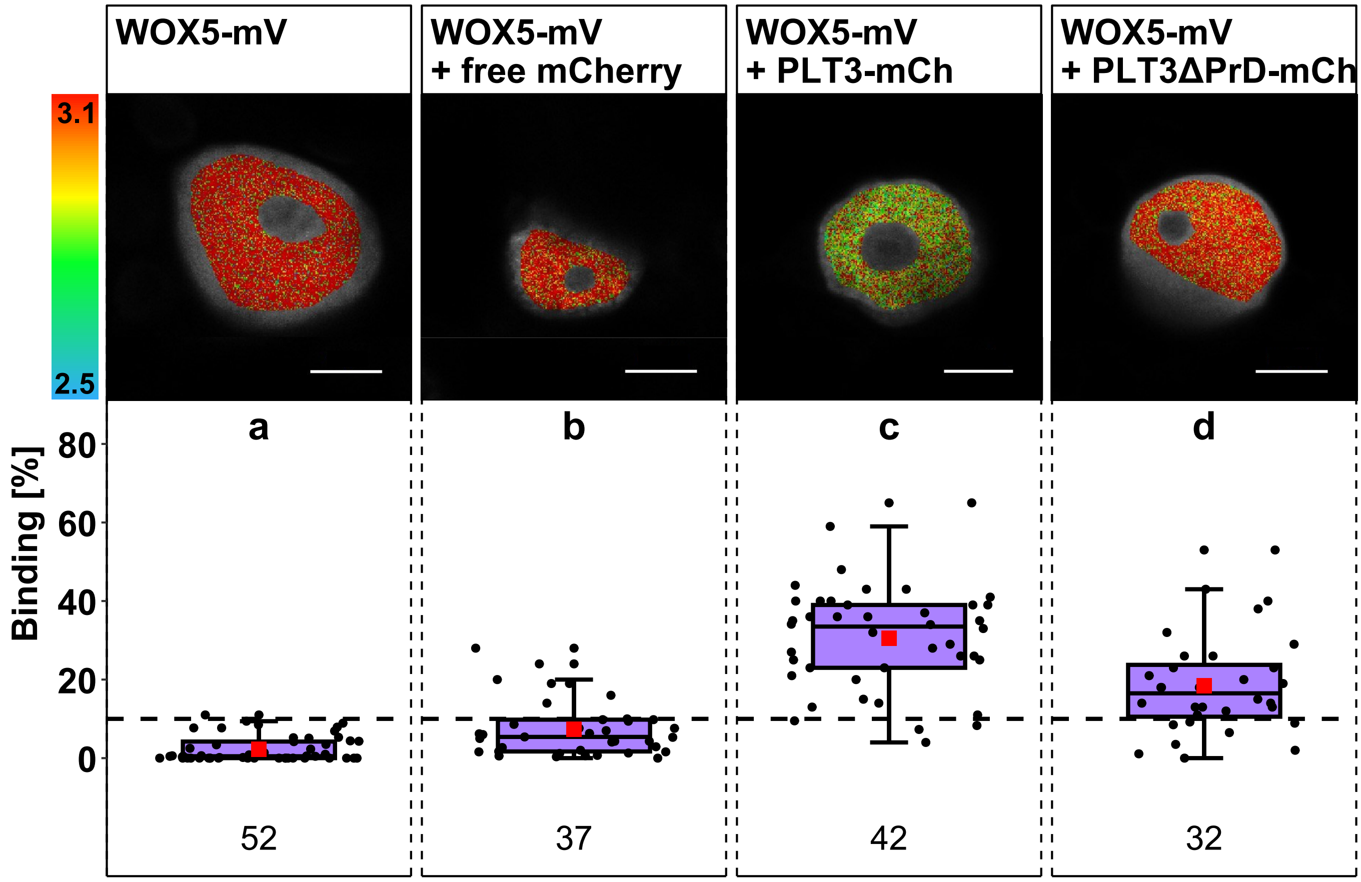

Appendix Figure S3. Interaction and affinity of PLT3 and WOX5 is altered upon loss of PLT3 PrDs.

Upper panel Representative images of fluorescence lifetime imaging microscopy (FLIM) measurements in *N. benthamiana* epidermal leaf cells after a pixel-wise multiexponential fit. The fluorescence lifetime of the donor WOX5-mV in the presence or absence of the indicated acceptor is color-coded: blue (2.5) refers to low fluorescence lifetime [in ns], red (3.1) indicates high fluorescence lifetime. Scale bars: 6 µm.

Lower panel Binding [%] (magenta) for WOX5-mV with or without co-expression of mCherry-NLS, PLT3-mCh or PLT3ΔPrD-mCh. Statistical groups were assigned after a non-parametric Kruskal Wallis with *post-hoc* Dunn's test ($\alpha = 0.05$, *p*-values were adjusted after Benjamini and Hochberg). The black dotted line indicates the Binding cut-off of 10 %. The numbers of analysed nuclei (biological replicates) are indicated below each sample and result from 2-5 technical replicates.

Data Information: Box = middle 50 % of data (= interquartile range (IQR)); whiskers = from IQR to min/max values, but at most 1.5 * IQR; line within box = median; data beyond the end of whiskers are ‚outliers‘ and are plotted independently. In addition, a jitter plot was used to visualize all data points individually.

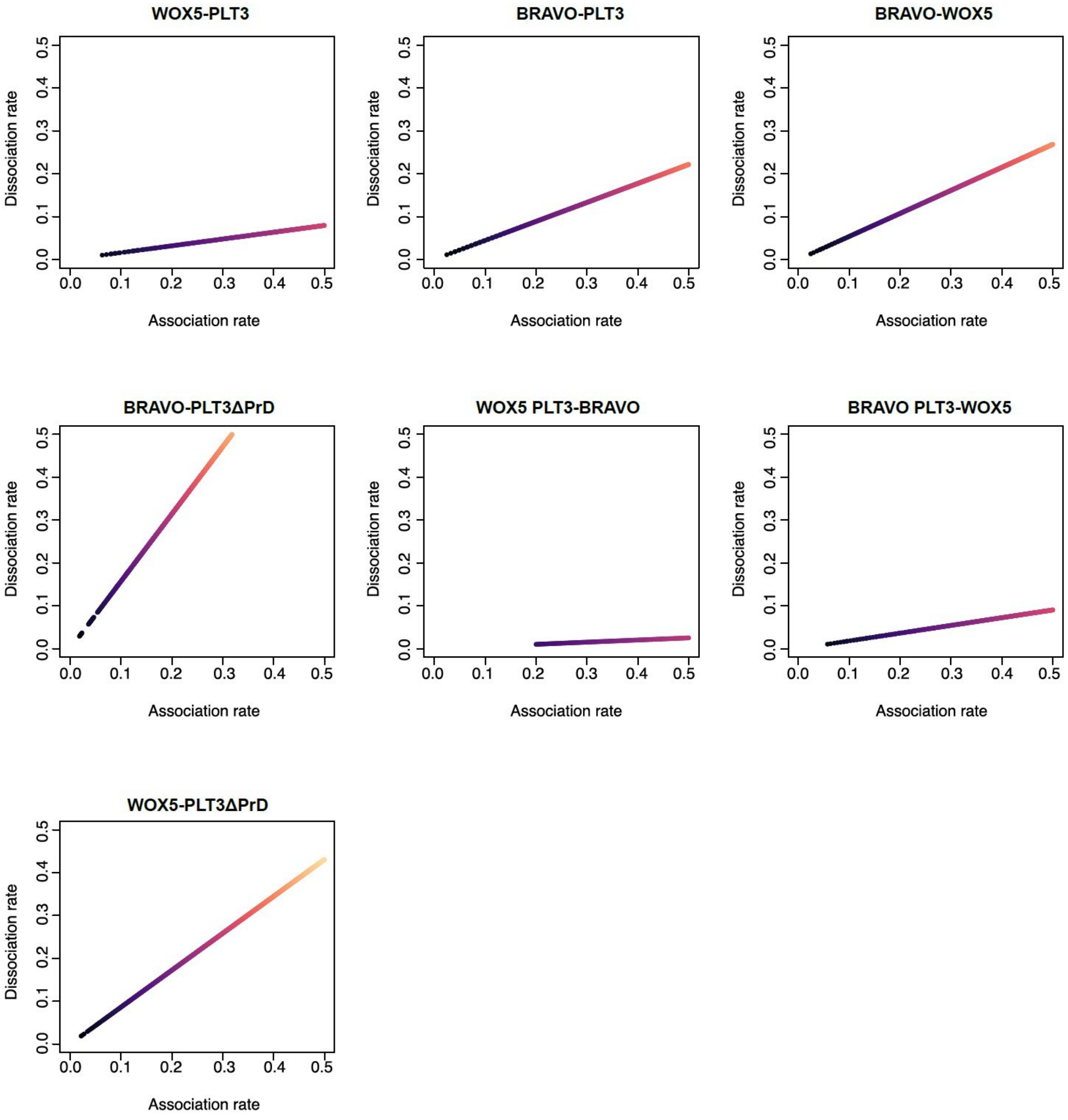

Appendix Figure S4. Association and dissociation parameters predicted for the heterodimers and trimeric complex modelled.

For each protein complex studied we show the combination of association and dissociation parameters (coloured area) that can produce the protein complex formation in agreement with the binding affinities described experimentally.

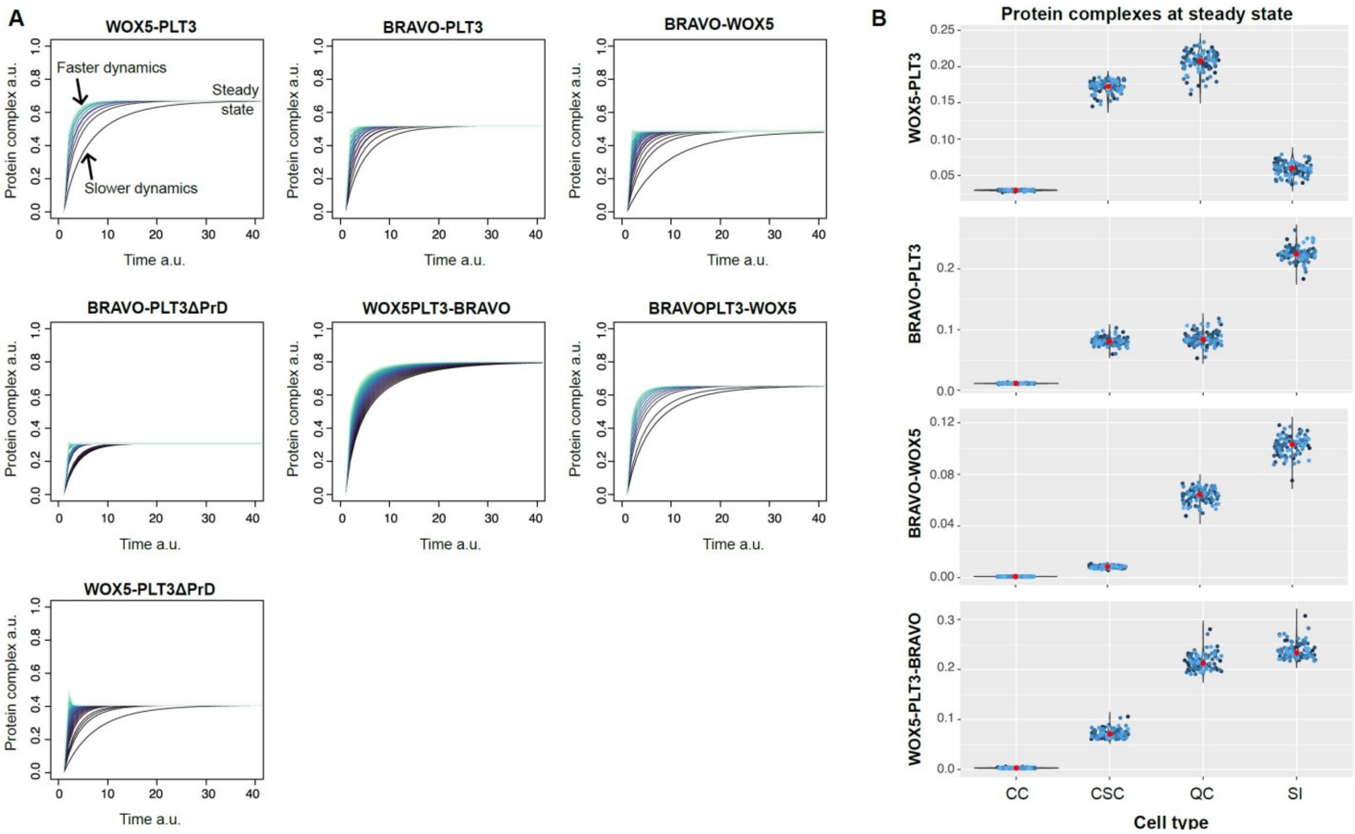

Appendix Figure S5. Robustness of the protein complex cell signatures.

A Temporal dynamics for the formation of each different complex using different parameter sets.

B Simulations of the formation of protein complexes in the cells of the root stem cell niche using 100 different association and dissociation parameter sets. The resulting levels of each simulation for WOX5-PLT3, BRAVO-PLT3, BRAVO-WOX5 and WOX5-PLT3-BRAVO in each cell type are shown, indicating they are robust to the specific parameters used in the simulations. Mean values shown in red.

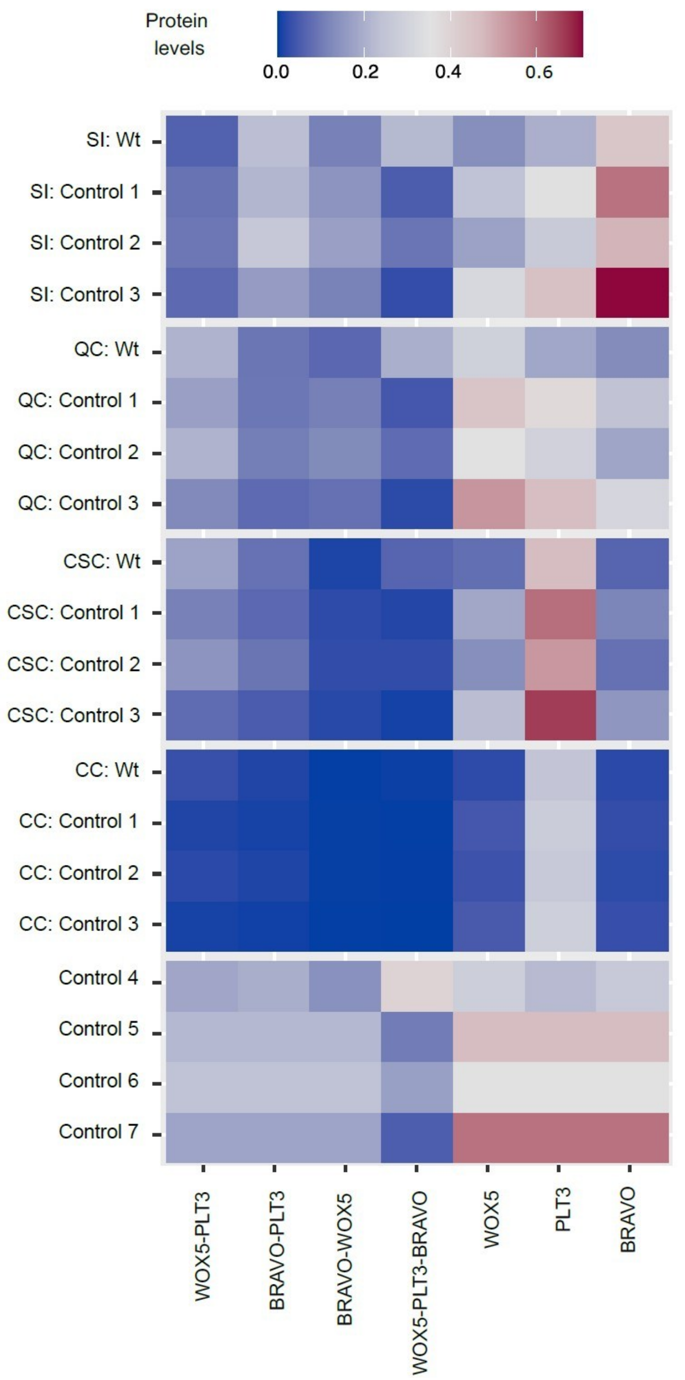

Appendix Figure S6. Controls for *in silico* prediction of protein complex signatures in the WT root SCN.

For Control 1-3, the experimentally determined protein abundances were used, combined with the assumptions that association and dissociation rates are equal, a higher association and a higher dissociation rate, respectively. For Control 4, experimentally determined association and dissociation rates were used in combination with equal protein abundances among all cell-types and TFs. Controls 5-7 combine equal protein levels with assumed association/dissociation rates from

Control 1-3, respectively. Heatmap showing the protein complexes and free protein in the cells of WT simulation. High concentrations are displayed in red, low concentration are displayed in blue. SI: stele initials; QC: quiescent center; CSC: columella stem cells; CC: columella cells.

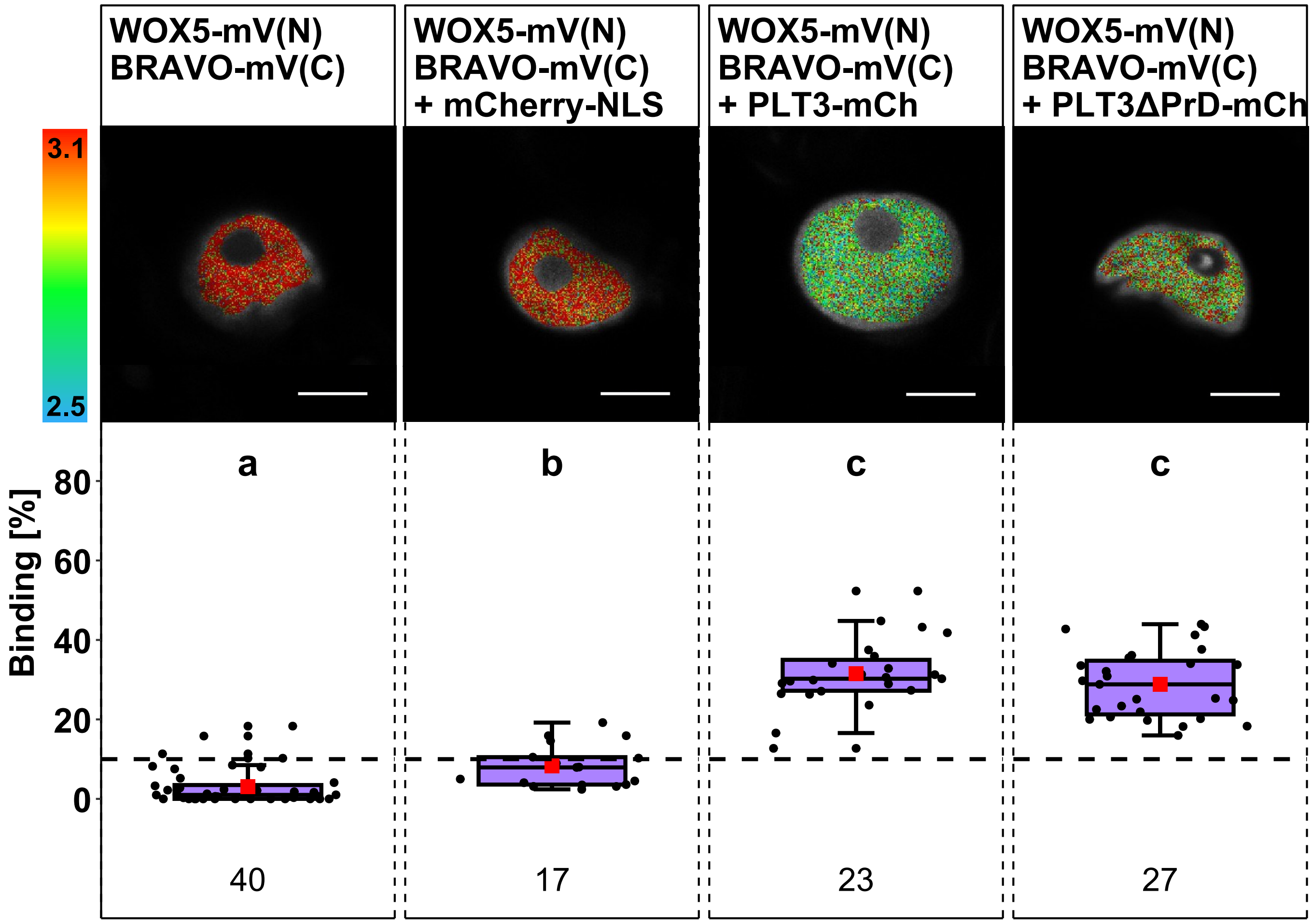

Appendix Figure S7. Trimeric complex formation of WOX5, BRAVO and PLT3 is only mildly affected upon loss of PLT3 PrDs.

Upper panel Representative images of fluorescence lifetime imaging microscopy (FLIM) measurements in *N. benthamiana* epidermal leaf cells after a pixel-wise multiexponential fit. The fluorescence lifetime of the donor WOX5-mV(N) BRAVO-mV(C) in the presence or absence of the indicated acceptor is color-coded: blue (2.5) refers to low fluorescence lifetime [in ns], red (3.1) indicates high fluorescence lifetime. Scale bars: 6 µm.

Lower panel Binding [%] (magenta) for WOX5-mV(N) BRAVO-mV(C) with or without co-expression of mCherry-NLS, PLT3-mCh or PLT3ΔPrD-mCh. Statistical groups were assigned after a non-parametric Kruskal Wallis with *post-hoc* Dunn's test (α = 0.05, *p*-values were adjusted after Benjamini and Hochberg). The black dotted line indicates the Binding cut-off of 10 %. The numbers of analysed nuclei (biological replicates) are indicated below each sample and result from 3-4 technical replicates.

Data Information: Box = middle 50 % of data (= interquartile range (IQR)); whiskers = from IQR to min/max values, but at most 1.5 * IQR; line within box = median; data beyond the end of whiskers are ‚outliers‘ and are plotted independently. In addition, a jitter plot was used to visualize all data points individually.

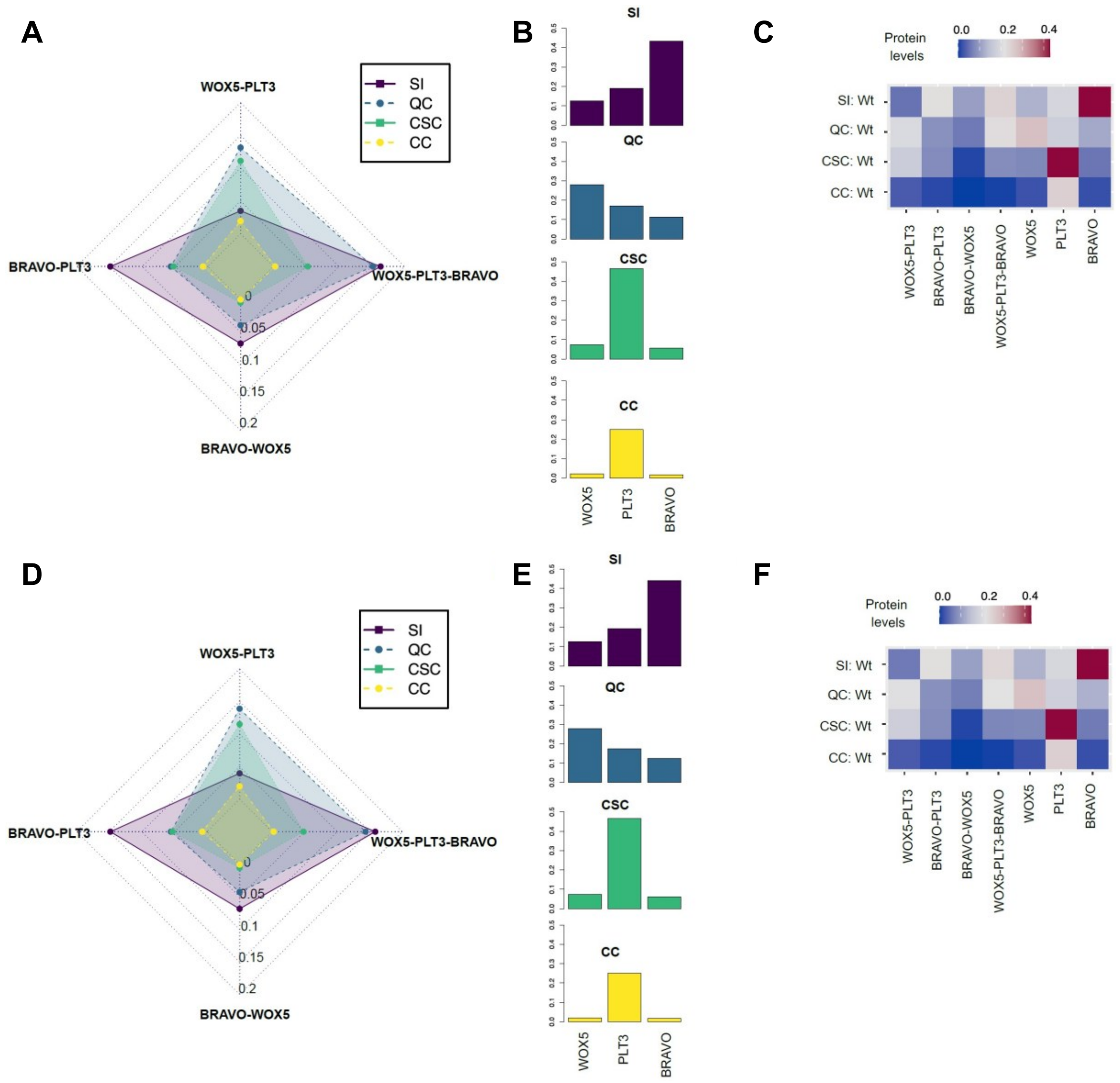

Appendix Figure S8. Control simulations to compare fast and slow dynamics of all parameter settings describing protein complex formation.

A, D Radar plot showing the levels of heterodimers and trimeric complex of WOX5, PLT3 and BRAVO formed in the SI (purple), QC (blue), CSC (green) and CC (yellow) for fast **(A)** or slow dynamics **(D)** of all parameter settings that describe protein complex formation. The radial axis shows the protein levels (in arbitrary units).

B, E Free WOX5, PLT3 and BRAVO protein in each of the simulated root SCN cells for fast **(B)** or slow dynamics **(E)** of all parameter settings that describe protein complex formation.

C, F Heatmap showing the protein complexes and free protein in the cells of WT simulation for fast **(C)** or slow dynamics **(F)** of all parameter settings that describe protein complex formation.

Appendix Table S1. List of primers used for cloning. Italic bases represent overhangs and *Bsa*I recognition sites necessary for GreenGate cloning.

Gene identifier	alias	Primer name	Orientation	Sequence 5'-3' orientation
Promoter modules				
AT5G17800	BRAVO	VS_GG_pBRAVO_F	F	*AAAGGTCTCAACCT*CCACTAACCATTTCGTAA
		VS_GG_pBRAVO_R	R	*AAAGGTCTCATGTT*GTTTCTGGTTTAGGGATTA
CDS in C modules				
AT1G19350	BES1	VS_GG_BES1D_CDS_F	F	*AAAGGTCTCAGGCTTA*ATGACGTCTGACGGAGCAAC
		VS_GG_BES1D_CDS_R	R	*AAAGGTCTCACTGA*ACTATGAGCTTTACCATTTCC
AT5G17800	BRAVO	RD_GG_BRAVO F	F	*AAAGGTCTCAGGCTTA*ATGAATCCAAATC
		RD_GG_BRAVO R	R	*AAAGGTCTCACTGA*GGAAGCTCCAAC
AT1G15750	TPL	VS_GG_TPL_CDS_F	F	*AAAGGTCTCAGGCTTA*ATGTCTTCTCTTAG
		VS_GG_TPL_CDS_R	F	*AAAGGTCTCACTGA*TCTCTGAGGCTG

Appendix Table S2. List of primers used for genotyping.

Gene ID	SALK ID	alias	Primer		
			Orientation	Name	Sequence 5'-3'
AT5G17800	SALK_062413	*bravo-2*	F	VS_bravo-2_F	TCCCTTAATCCCTAAACCCAGC
			R	VS_bravo-2_R	CCTGATGCAAGGGTACTATCG
AT3G11260	SALK_038262	*wox5-1*	F	GK_WOX5 F	AAACAGTTGAGGACTTTACATCTGA
			R	WOX5 R	CGGATAATATGTCATAATTCAAAAT
AT5G10510	SALK_127417	*plt3-1*	F	GK_PLT3L	TTGTGATTTGCCATTGACTAAAGGT
			R	GK_PLT3R	GAAAACAGTCCAATGGTCTCACATC

Appendix Table S3. List of entry vectors used for GreenGate cloning.

Name	Module (Backbone)	Insert	Reference
pGGB002	B	Omega element	(Lampropoulos *et al,* 2013)
pGGD007	D	Linker-NLS	
pGGE009	E	UBIQUITIN 10 terminator	
pGGF002	F	BASTA resistance	
pGGG001	G	Adapter	
pGGG002	G	Adapter	
pGGM000	M	Empty intermediate vector	
pGGN000	N	Empty intermediate vector	
pGGZ001	Z	Empty destination vector	
pRD42	C	mVenus	(Burkart *et al,* 2022)
pRD43	D	mVenus	
pRD53	D	mCherry	
pRD45	A	*WOX5* promoter	
pRD40	C	WOX5 CDS	
pRD41	C	PLT3 CDS	
pRD65	B	Glucocorticoid receptor	
pRD101	C	PLT3ΔPrD	
pPD161	A	Ubi-XVE oLexA-35S	(Denninger *et al,* 2019)
pVS125	A	*BRAVO* promoter	This study
pRD135	C	BRAVO CDS	
pVS191	C	BES1D	
pVS84	C	TPL	
pJM81	D	mVenus(N)	(Maika *et al,* 2023)
pJM82	D	mVenus(C)	
pBLAD011	D	mTurquoise2	

Appendix Table S4. List of expression vectors for stable transformation of *A. thaliana* or transient transformation of *N. benthamiana* generated in this study.

Plasmid ID	Construct	GreenGate module							Resistance
		A	B	C	D	E	F	Z	
pVS133	pBRAVO:BRAVO-mV	*BRAVO* promoter	Ω element (pGGB002)	BRAVO	mVenus	UBQ10 terminator (pGGE009)	BASTA R (pGGF002)	pGGZ001	Spec
pVS139	Inducible BES1D-mVenus	Ubi-XVE oLexA-35S	Ω element (pGGB002)	BES1D	mVenus	UBQ10 terminator (pGGE009)	BASTA R (pGGF002)	pGGZ001	Spec
pVS140	Inducible BES1D-mCherry	Ubi-XVE oLexA-35S	Ω element (pGGB002)	BES1D	mCherry	UBQ10 terminator (pGGE009)	BASTA R (pGGF002)	pGGZ001	Spec
pVS141	Inducible BRAVO-mVenus	Ubi-XVE oLexA-35S	Ω element (pGGB002)	BRAVO	mVenus	UBQ10 terminator (pGGE009)	BASTA R (pGGF002)	pGGZ001	Spec
pVS142	Inducible BRAVO-mCherry	Ubi-XVE oLexA-35S	Ω element (pGGB002)	BRAVO	mCherry	UBQ10 terminator (pGGE009)	BASTA R (pGGF002)	pGGZ001	Spec
pVS143	Inducible TPL-mCherry	Ubi-XVE oLexA-35S	Ω element (pGGB002)	TPL	mCherry	UBQ10 terminator (pGGE009)	BASTA R (pGGF002)	pGGZ001	Spec
pVS85	Inducible TPL-mVenus	Ubi-XVE oLexA-35S	Ω element (pGGB002)	TPL	mVenus	UBQ10 terminator (pGGE009)	BASTA R (pGGF002)	pGGZ001	Spec
pVS154	Inducible WOX5-mVenus(N)	Ubi-XVE oLexA-35S	Ω element (pGGB002)	WOX5	mVenus(N)	UBQ10 terminator (pGGE009)	-	pGGM000	Kan
pVS156	Inducible PLT3-mVenus(C)	Ubi-XVE oLexA-35S	Ω element (pGGB002)	PLT3	mVenus(C)	UBQ10 terminator (pGGE009)	BASTA R (pGGF002)	pGGN000	Kan
pVS163	Inducible WOX5-mVenus(N)/ Inducible PLT3-mVenus(C)	pVS154 + pVS156						pGGZ001	Spec
pVS167	Inducible nuclear localized mCherry	Ubi-XVE oLexA-35S	Ω element (pGGB002)	mCherry (pGGC015)	linker-NLS (pGGD007)	UBQ10 terminator (pGGE009)	BASTA R (pGGF002)	pGGZ001	Spec
pVS180	Inducible BRAVO-mVenus(N)	Ubi-XVE oLexA-35S	Ω element (pGGB002)	BRAVO	mVenus(N)	UBQ10 terminator (pGGE009)	-	pGGM000	Kan

pVS233	Inducible BRAVO-mVenus(N)/ Inducible PLT3-mVenus(C)	pVS180 + pVS156						pGGZ001	Spec
pVS288	pWOX5:GR-PLT3-mTurquoise2	*WOX5* promoter	GR	PLT3	mT2	UBQ10 terminator (pGGE009)	BASTA R (pGGF002)	pGGZ001	Spec
pVS289	pWOX5:GR-PLT3ΔPrD-mTurquoise2	*WOX5* promoter	GR	PLT3ΔPrD	mT2	UBQ10 terminator (pGGE009)	BASTA R (pGGF002)	pGGZ001	Spec
pVS182	Inducible BRAVO-mVenus(C)	Ubi-XVE oLexA-35S	Ω element (pGGB002)	BRAVO	mVenus(C)	UBQ10 terminator (pGGE009)	BASTA R (pGGF002)	pGGN000	Kan
pVS188	Inducible WOX5-mVenus(N)/ Inducible BRAVO-mVenus(C)	pVS154 + pVS182						pGGZ001	Spec

Appendix Table S5. List of Arabidopsis mutants and transgenic lines used in this study.

Gene ID	Alias	Reference
AT5G17800	*bravo-2*	(Vilarrasa-Blasi *et al,* 2014)
	Col-0, pBRAVO:BRAVO-mVenus	This study by dipping
AT5G10510	*plt3-1*	(Galinha *et al,* 2007)
	Col-0, pPLT3:PLT3-mVenus	(Burkart *et al,* 2022)
AT5G17800, AT5G10510	*bravo-2, plt3-1*	This study by crossing of *bravo-2* and *plt3-1*
AT3G11260	*wox5-1*	(Burkart *et al,* 2022)
	Col-0, pWOX5:WOX5-mVenus	
AT5G10510, AT3G11260	*plt3-1, wox5-1*	(Burkart *et al,* 2022)
	plt3-1, pWOX5:GR-PLT3-mTurquoise2	This study by dipping
	plt3-1, pWOX5:GR-PLT3ΔPrD-mTurquoise2	This study by dipping
AT5G17800, AT3G11260	*bravo-2, wox5-1*	(Betegón-Putze *et al,* 2021)
AT5G17800, AT5G10510, AT3G11260	*bravo-2, plt3-1, wox5-1*	This study by crossing of *bravo-2* and *plt3-1, wox5-1*

Appendix Table S6. Fluorescence intensities of *pPLT3:PLT3-mV, pBRAVO:BRAVO-mV* and *pWOX5:WOX5-mV* translational reporter in different cell types corresponding to Fig 1, Fig 5 and Fig 7.

Fluorescence intensity	Date	Cell type			
		SI	QC	CSC	CC
pPLT3:PLT3-mV	28.11.23	30360.17	37523.45	48567.14	17482.71
		19709.9	18540.11	22274.4	15590.86
		19782.11	16327.93	26233.66	6335.124
		24665.88	22914.56	22039.49	13233.82
		16336.64	14473.61	16777.05	7263.823
		20659.51	17532.76	19131.18	9199.955
		48822.24	37769.87	55574.26	5490.301
		42071.03	23881.81	28888.19	6474.87
		31306.43	28682.1	43367.48	26115.04
		26035.98	24754.96	37376.88	5407.617
	**AV**	27974.99	24240.11	32022.97	11259.41
	**SD**	9945.883	7853.973	12737.42	6457.178
	08.12.23	13458.53	12022.34	10693.72	3079.556
		11382.8	19661.37	23237.77	2543.246
		31222.01	28196.38	24778.53	1576.29
		28921.69	24819.56	21161.82	6278.363
		25231.6	12692.34	9225.459	9485.656
		14733.96	15308.52	30900.44	19996.52
		13009.25	19128.18	18887.84	1805.671
		17399.32	20006.95	19149.94	6983.197
		10872.27	8227.07	12223.45	1741.223
		21828.1	21030.97	24277.79	2664.413
	**AV**	18805.95	18109.37	19453.67	5615.413
	**SD**	7111.341	5781.51	6587.363	5426.973
	28.12.23	36705.69	30759.92	32179.98	13431.11
		23201.13	22910.14	26746.3	12992.69
		14505.16	16790.7	14650.75	10778.34
		25326.28	16618.53	15902.32	7739.259
		22976.1	22252.79	13183.53	15139.32
		26289.13	34416.08	46981.29	13944.07
		23456.61	45225.47	45902.98	7467.853
		19359.14	19409.83	23114.97	23172.93
	**AV**	**23977.41**	**26047.93**	**27332.76**	**13083.19**
	**SD**	**5938.235**	**9373.698**	**12570.98**	**4640.888**
	**Overall AV**	**23267.05**	**22671.05**	**25904.29**	**9224.714**
	**Overall SD**	**8745.45**	**8164.877**	**11578.14**	**6400.249**
	**n**	**28**	**28**	**28**	**28**
pWOX5:WOX5-mV	28.11.23	29201.12	45834.2	10795.79	896.549
		13869.69	21386.9	8486.974	1496.997
		19027.65	28070.95	7576.267	779.7283
		14906.16	27262.89	9271.191	685.0723
		15691.41	27641.05	6953.646	687.679

		17316.21	20991.05	10378.01	705.1347
		11098.75	30414.8	9765.741	766.4405
		29355.06	22623.3	7448.115	882.8665
		18739.97	22616.32	9814.987	1002.133
		13616.57	17752.29	12525.81	7864.905
	**AV**	18282.26	26459.37	9301.653	1576.75
	**SD**	5953.816	7460.556	1638.761	2108.523
	**08.12.23**	11408.16	12757.81	7483.419	532.5793
		14144.85	17938.5	8482.929	958.597
		18676.1	20097.02	12921.63	838.317
		18215.11	22406.33	10450.67	730.0777
		12329.53	17780.14	8244.796	3459.093
		32552.77	32325.28	15897.64	8156.247
		18057.62	34627.05	17197.87	833.5587
		20081.02	33351.82	12183.4	2112.58
		16962.96	27887.07	13940.99	854.592
		11985.38	14764.86	6683.364	731.142
	**AV**	17441.35	23393.59	11348.67	1920.678
	**SD**	5841.4	7655.123	3466.346	2246.346
	**28.12.23**	18809.39	21126.97	13228.5	969.778
		18091.15	21354.1	13364.58	661.5485
		13782.29	25015.66	7060.959	837.938
		11749.74	32733.76	10815.74	2313.319
		16980.22	19147.65	10490.34	719.408
		16397.84	28137.24	15536.71	1107.079
		17359.42	26238.14	10937.61	680.671
		16533.92	36878.85	15549.16	7561.397
	**AV**	**16213**	**26329.05**	**12122.95**	**1856.392**
	**SD**	**2183.395**	**5708.609**	**2682.338**	**2214.819**
	**Overall AV**	**16790.78**	**25132.08**	**11061.31**	**1684.39**
	**Overall SD**	**5507.95**	**7039.065**	**3596.461**	**2105.804**
	**n**	**28**	**28**	**28**	**28**
pBRAVO:BRAVO-mV	**28.11.23**	33296	16940.47	4615.491	852.569
		32465.25	12767.84	8112.653	989.9733
		36508.05	19942.11	14826.77	1133.772
		31225.91	18182.72	8115.524	919.5017
		40549.32	20651.28	8776.193	1081.686
		40143.53	29515.55	8153.343	1102.827
		19431.36	10567.79	3988.657	695.031
		29660.65	12654.5	4704.077	1479.854
		28870.52	16931.86	3053.96	872.8765
		31988.38	16361	8163.813	1257.115
	**AV**	32413.9	17451.51	7251.048	1038.52
	**SD**	5777.106	5065.289	3233.365	213.1088
	**08.12.23**	17139.23	12128.37	3986.173	845.2763
		35034.8	21779.82	10615.84	1246.433
		32923.36	12882.26	6763.008	946.799

		19276.64	14514.07	9689.346	906.56
		25266.62	12037.34	6404.131	734.922
		41025.65	18926.21	7023.33	1303.169
		24780.18	10123.88	3126.593	844.1405
		39899.04	18246.41	10925.48	1296.336
		22975.67	13452.88	11498.59	1244.351
		31024.35	13617	9103.976	1312.867
	**AV**	28934.55	14770.82	7913.646	1068.085
	**SD**	7890.066	3481.702	2766.423	219.4884
	**28.12.23**	46147.85	26669.37	16433.59	1442.835
		49948.21	7967.577	2823.43	770.814
		39406.01	34224.94	6801.504	1497.628
		41437.48	16972.48	5323.483	1414.79
		25584.47	9576.138	2069.954	805.6805
		40467.97	9512.593	5478.113	1207.251
		42555.79	10385.78	5205.178	1349.889
		30363.89	13326.26	9106.051	1152.85
	**AV**	**39488.96**	**16079.39**	**6655.162**	**1205.217**
	**SD**	**7454.075**	**8898.174**	**4222.165**	**264.0782**
	**Overall AV**	**33047.28**	**15892.74**	**6833.518**	**1058.715**
	**Overall SD**	**7897.687**	**6132.839**	**3588.269**	**257.7053**
	**n**	**28**	**28**	**28**	**28**

AV: average, SD: standard deviation, n: number of observations.

Appendix Table S7. Average number of QC divisions and CSC layers per root related to Appendix Fig S1.

Genotype	Average number of QC divisions per root	Average number of CSC layers per root	Number of analysed roots
bravo-2	1.75	0.7917	24
bravo-2, pBRAVO:BRAVO-mV	0.33	1.2592	27

Appendix Table S8. Ratio of periclinal cell division planes in the QC related to Appendix Fig S1.

Genotype	Periclinal cell division planes in the QC [%]	Number of analysed roots
bravo-2	92	24
bravo-2, pBRAVO:BRAVO-mV	4	27

Appendix Table S9. Average number of QC divisions and CSC layers per root related to Fig 2 and Fig EV1.

Genotype	Average number of QC divisions per root	Average number of CSC layers per root	Number of analysed roots
Col-0	0.54	1.28	99
bravo-2	1.30	1.12	73
plt3-1	1.39	1.29	75
wox5-1	1.91	0.59	74
bravo, plt3	1.53	0.73	30
bravo, wox5	2.84	0.10	58
plt3, wox5	2.77	0.26	57
bravo, plt3, wox5	3.15	0.16	55

Appendix Table S10. Ratio of periclinal cell division planes in the QC related to Fig EV1.

Genotype	Periclinal cell division planes in the QC [%]	Number of analysed roots
Col-0	4	99
bravo-2	85	73
plt3-1	43	75
wox5-1	62	78
bravo, plt3	77	30
bravo, wox5	84	59
plt3, wox5	79	57
bravo, plt3, wox5	85	55

Appendix Table S11. Root length of different *Arabidopsis thaliana* genotypes at 10 DAG in mm related to Appendix Fig S2.

**Date**	**Root**	**Genotype**							
		Col-0	***bravo-2***	***plt3-1***	***wox5-1***	***bravo, plt3***	***bravo, wox5***	***plt3, wox5***	***bravo, plt3, wox5***
**06.09.2024**	**#1**	37.5	50.2	50.1	48.8	65.2	57.7	50.7	51.3
	**#2**	60.1	63.4	57.6	55.3	60.8	52.0	54.5	58.2
	**#3**	56.4	37.9	50.6	56.6	67.0	63.5	64.6	52.3
	**#4**		61.7	48.1	49.0	54.9	34.3	60.7	47.2
	**#5**		58.7	48.6	40.7	61.2	59.4	52.2	33.9
	**#6**		57.2	52.6	59.3	44.2	53.7	50.5	58.8
	**#7**		59.3	40.7	58.1	50.4	53.8		58.5
	**#8**		61.2		60.3	50.9			
	**#9**								
	**#10**								

**09.09.2024**	**#1**	57.2	59.2	52.5	61.1	65.2	63.7	62.2	51.0
	**#2**	59.1	54.2	59.6	57.5	64.7	45.8	57.4	41.3
	**#3**	51.4	60.9	53.5	58.5	58.2	51.3	47.5	49.0
	**#4**	47.9	45.3	39.8	58.2	49.4	42.7	46.7	41.4
	**#5**	37.2	57.5	48.5	57.3	54.7		56.4	42.5
	**#6**	51.2	52.7	56.2		52.4		51.1	47.3
	**#7**	43.5	56.6			53.8			47.0
	**#8**		61.1			49.2			47.3
	**#9**		62.2			55.5			51.6
	**#10**								
**26.09.2024**	**#1**	54.1	57.2	53.8	57.4	54.5	57.8	50.9	51.2
	**#2**	53.7		50.9	51.2	57.7	53.3	51.6	48.6
	**#3**	54.5		54.7	48.4	67.5	50.0	45.5	53.9
	**#4**	57.0		46.7	47.6	56.8	46.3	51.5	54.7
	**#5**	58.9		59.8	51.8	59.5	45.1	56.5	52.1
	**#6**	49.7		57.3	50.3	62.6		50.1	46.3
	**#7**	53.4		58.9		66.6		42.3	44.3
	**#8**	55.9		59.7		54.5		52.1	42.9
	**#9**			57.1				45.5	39.6
	**#10**			47.5				57.7	
	**#11**							57.8	
	**#12**							43.9	
**n**		**18.0**	**18.0**	**23.0**	**19.0**	**25.0**	**16.0**	**24.0**	**25.0**
**AV**		**52.1**	**56.5**	**52.4**	**54.1**	**57.5**	**51.9**	**52.5**	**48.5**
**SD**		**6.6**	**6.3**	**5.5**	**5.3**	**6.3**	**7.6**	**5.7**	**6.1**

N: number of observations, AV: average, SD: standard deviation.

Appendix Table S12. Measured FRET efficiency and Binding values related to Fig 3, Fig 5 and Fig 7.

**Date**	**BRAVO-mV**		**BRAVO-mV mCherry-NLS**		**BRAVO-mV PLT3-mCh**		**BRAVO-mV BES1D-mCh**		**BRAVO-mV WOX5-mCh**		**BRAVO-mV TPL-mCh**	
	**FRET E**	**Binding**	**FRET E**	**Binding**	**FRET E**	**Binding**	**FRET E**	**Binding**	**FRET E**	**Binding**	**FRET E**	**Binding**
**20.12.19**	80.00	3.00			54.00	34.00						
	80.00	1.70			51.00	33.00						
	80.00	2.40			56.00	35.00						
	80.00	7.10			54.00	30.00						
	80.00	5.30			60.00	40.00						
	80.00	2.50			53.00	45.00						
	80.00	8.00			57.00	35.00						
	80.00	8.90			53.00	40.00						
	80.00	5.10			58.00	32.00						
	78.00	8.00			59.00	34.00						

04.02.20	9.88	-11.00					80.00	9.20				
	9.88	-17.00					0.97	7.00				
	17.00	7.40					0.66	37.00				
	10.00	-0.30					0.90	13.00				
	71.00	6.30					0.49	34.00				
	80.00	6.90					0.93	15.00				
	80.00	8.20					1.10	22.00				
	9.88	-23.00					0.64	40.00				
	67.00	4.30					1.30	9.05				
	16.00	5.00										
17.02.20	80.00	2.50	45.00	11.00			48.00	29.00	58.00	9.60	43.00	24.00
	80.00	0.51	43.00	8.41			48.00	25.00	55.00	27.00	39.00	27.00
	41.00	8.20	63.00	8.90			46.00	35.00	64.00	14.00	37.00	30.00
	80.00	2.40	50.00	15.00			47.00	19.00	61.00	14.00	46.00	28.00
	10.10	-11.00	53.00	14.00			41.00	29.00	56.00	33.00	40.00	29.00
	75.00	2.20					42.00	30.00	58.00	19.00	43.00	30.00
	80.00	1.00					45.00	33.00	59.00	27.00	40.00	34.00
	80.00	4.10					45.00	47.00	51.00	7.30	42.00	32.00
	80.00	1.40					45.00	41.00	64.00	7.00	49.00	20.00
	80.00	6.70					48.00	49.00	65.00	11.00	38.00	29.00
							41.00	36.00	40.00	24.00	48.00	27.00
							45.00	41.00	49.00	27.00	44.00	32.00
							48.00	42.00	52.00	31.00	77.00	73.00
							45.00	37.00	50.00	39.00	43.00	37.00
							44.00	39.00	55.00	61.00	43.00	34.00
17.08.20	80.00	6.40	80.00	12.00	74.00	8.70			79.00	15.00	59.00	16.00
	80.00	8.20	66.00	9.60	61.00	28.00			66.00	10.00	57.00	18.00
	80.00	11.00	68.00	11.00	80.00	10.00			65.00	17.00	55.00	23.00
	80.00	11.00	80.00	8.90	80.00	8.70			56.00	24.00	56.00	22.00
	80.00	14.00	80.00	13.00	73.00	10.70			55.00	26.00	56.00	20.00
	80.00	5.50	77.00	8.70	77.00	12.00			63.00	14.00	50.00	26.00
	80.00	6.40	80.00	12.00	80.00	9.60					76.00	10.00
	80.00	3.50	78.00	15.00	62.00	16.00					48.00	25.00
	80.00	6.50			55.00	24.00					53.00	20.80
	80.00	3.20			52.00	25.00					52.00	23.00
27.06.22	35.00	1.10	63.00	1.40	49.00	14.00	47.00	13.00	44.00	13.00	44.00	17.00
	80.00	3.50	80.00	3.90	45.00	40.00	45.00	21.00	59.00	9.10	43.00	25.00
	78.00	5.00	63.00	2.40	43.00	26.00	44.00	19.00	43.00	18.00	38.00	29.00
	80.00	-0.15	54.00	2.10	48.00	19.00	39.00	18.00	48.00	53.00	45.00	20.00
	9.92	-2.40	61.00	5.70	47.00	32.00	43.00	29.00	45.00	27.00	40.00	30.00
	65.00	3.10	37.00	4.60	46.00	39.00	45.00	25.00	52.00	56.00	41.00	23.00
	79.00	3.80			43.00	32.00	45.00	23.00	55.00	9.10	37.00	27.00
	73.00	1.20			47.00	42.00	56.00	15.00	56.00	13.00	41.00	17.00
	9.92	-16.00			41.00	44.00	45.00	9.50	45.00	30.00	39.00	30.00
	9.92	-15.00			39.00	40.00	42.00	41.00	42.00	8.40	35.00	27.00
**N**	**50.00**	**50.00**	**19.00**	**19.00**	**30.00**	**30.00**	**34.00**	**34.00**	**31.00**	**31.00**	**35.00**	**35.00**
**AV**	**63.49**	**2.33**	**64.26**	**8.82**	**56.57**	**27.96**	**35.76**	**27.40**	**55.16**	**22.37**	**46.77**	**26.71**
**SD**	**27.50**	**7.43**	**13.81**	**4.26**	**11.94**	**11.69**	**20.41**	**11.85**	**8.45**	**14.08**	**9.66**	**9.82**

N: number of observations, AV: average, SD: standard deviation.

Appendix Table S13. FRET efficiencies and Binding related to Fig 4 and Fig 5.

Date	WOX5-mV(N) PLT3-mV(N)		WOX5-mV(N) PLT3-mV(N) mCherry-NLS		WOX5-mV(N) PLT3-mV(N) BRAVO-mCh		WOX5-mV(N) PLT3-mV(N) BES1D-mCh		WOX5-mV(N) PLT3-mV(N) TPL-mCh	
	**FRET E**	**Binding**	**FRET E**	**Binding**	**FRET E**	**Binding**	**FRET E**	**Binding**	**FRET E**	**Binding**
**16.03.2020**	80.00	4.90			55.00	23.00	59.00	11.00	39.00	33.00
	80.00	6.50			50.00	52.00	53.00	18.00	39.00	15.00
	80.00	68.00			51.00	42.00	43.00	29.00	44.00	25.00
	80.00	-2.30			47.00	34.00	46.00	19.00	34.00	34.00
	10.10	-7.00			48.00	48.00	55.00	15.00	38.00	30.00
	80.00	8.90			46.00	45.00	61.00	33.50	40.00	26.00
	80.00	6.00			45.00	38.00	42.00	20.00	49.00	25.00
	78.00	8.80			52.00	50.00	48.00	24.00	48.00	18.00
	79.00	4.50			49.00	39.00	45.00	26.00	43.00	22.00
	80.00	5.10			46.00	36.00	43.00	33.00	39.00	31.00
							51.00	35.00	43.00	34.00
							44.00	39.00	48.00	29.00
							44.00	23.00	44.00	51.00
							46.00	28.00	48.00	20.00
									41.00	29.00
**18.12.2020**	9.96	-18.00	65.00	4.70	53.00	43.00	57.00	7.20	33.00	16.00
	9.96	-11.00	63.00	7.00	51.00	45.00	43.00	15.00	34.00	26.00
	80.00	-4.20	61.00	5.20	51.00	19.00	41.00	15.00	32.00	24.00
	9.96	-4.70	51.00	8.80	43.00	21.00	49.00	12.00	37.00	26.00
	9.96	-7.60	48.00	5.00	45.00	30.00	41.00	28.00	41.00	13.00
	73.00	2.60			48.00	48.00	45.00	13.00	33.00	28.00
	80.00	4.20			48.00	38.00	46.00	7.80	39.00	21.00
	54.00	2.20			51.00	25.00	55.00	6.05	41.00	14.00
	71.00	3.20			49.00	27.00	44.00	15.00	27.00	10.00
	18.00	3.70			45.00	36.00	39.00	10.00	35.00	20.00
							42.00	14.00		
							50.00	14.00		
							37.00	24.00		
							50.00	12.00		
							39.00	12.00		
**21.12.2020**	9.95	-15.00	12.00	-2.70	53.00	42.00	42.00	18.00	39.00	17.00
	9.95	-5.20	9.95	-9.10	51.00	48.00	41.00	13.00	31.00	29.00
	10.00	-3.60	10.00	4.30	46.00	34.00	56.00	6.90	43.00	19.00
	48.00	-0.40	51.00	5.20	47.00	25.00	44.00	22.00	45.00	18.00
	69.00	2.60	63.00	1.00	51.00	12.00	42.00	17.00	55.00	9.30
	17.00	8.30			46.00	18.00	39.00	16.00	42.00	14.00
	57.00	5.50			51.00	53.00	36.00	12.00	36.00	31.00
	9.95	-2.00			46.00	40.00	45.00	20.00	37.00	24.00

	9.95	-8.30			47.00	40.00	40.00	20.00	41.00	15.00
	9.95	-7.00			50.00	38.00	37.00	23.00	36.00	19.00
							41.00	16.00		
							38.00	26.00		
**AV**	**46.46**	**1.62**	**43.40**	**2.94**	**48.70**	**36.30**	**45.34**	**18.74**	**39.83**	**23.29**
**SD**	**31.88**	**14.13**	**22.14**	**5.01**	**2.88**	**10.73**	**6.24**	**8.03**	**5.75**	**8.29**
**N**	**30.00**	**30.00**	**10.00**	**10.00**	**30.00**	**30.00**	**41.00**	**41.00**	**35.00**	**35.00**

N: number of observations, AV: average, SD: standard deviation.

Appendix Table S14. FRET efficiencies and Binding related to Fig EV2 and Fig 7.

**Date**	**BRAVO-mV(N) PLT3-mV(N)**		**BRAVO-mV(N) PLT3-mV(N) mCherry-NLS**		**BRAVO-mV(N) PLT3-mV(N) WOX5-mCh**		**BRAVO-mV(N) PLT3-mV(N) BES1D-mCh**		**BRAVO-mV(N) PLT3-mV(N) TPL-mCh**	
	**Binding**	**FRET E**	**Binding**	**FRET E**	**Binding**	**FRET E**	**Binding**	**FRET E**	**Binding**	**FRET E**
**03.07.23**	0.00	10.17	0.00	8.47	31.40	44.41	7.10	10.17	11.00	49.15
	0.00	80.03	2.00	80.03	40.29	42.37	0.00	10.17	16.49	31.53
	2.35	80.03	0.00	8.47	35.98	42.81	0.00	10.17	1.46	80.03
	0.00	10.17	0.00	80.00	27.89	46.78	0.00	79.66	22.00	13.22
	0.00	80.03	0.00	10.17	30.09	38.98	0.00	10.17	25.10	14.58
	0.66	10.17					2.50	80.03	8.20	10.17
	1.33	10.17							7.02	10.17
**05.07.23**	0.00	80.00	2.51	79.97	26.61	46.67	6.72	43.33		
	0.00	10.00	0.00	10.00	26.49	40.67	11.10	26.67		
	0.00	10.00	10.60	15.67	8.29	51.00	16.37	31.67		
	0.00	79.97	4.10	60.00	23.92	33.33	18.80	38.33		
	0.00	10.00			36.81	46.53	13.05	35.67		
	14.20	10.00			43.11	43.00				
	1.80	10.33			8.91	63.33				
	0.00	10.00								
**04.12.23**	0.00	10.26	5.34	14.24	25.03	36.75	5.25	80.03	22.54	31.46
	1.85	30.46	5.97	25.17	50.54	48.01	13.14	35.43	19.53	36.09
	4.00	17.22	3.49	80.03	42.89	45.03	15.10	25.17	34.21	24.83
	9.00	10.26	2.99	10.26	27.60	42.72	4.95	40.40	35.26	26.16
	0.00	10.26	5.00	80.03	53.65	51.32	19.30	35.43	15.13	42.05
	1.06	27.15			45.49	45.43	17.91	28.81	21.90	32.45
	9.30	11.59			12.83	46.69	15.72	49.34	31.38	27.15
	0.00	80.13			19.65	45.36	14.77	45.36	25.12	31.79
	8.80	26.82			29.73	43.71	15.30	37.09	24.00	37.75
	1.87	80.03			36.98	44.70	11.88	37.75	34.16	29.80
**16.12.23**	0.00	79.93	5.30	10.03	33.80	48.16	11.34	39.80	17.02	56.52
	0.00	10.03	4.90	79.97	20.27	45.48	9.20	24.75	22.69	57.19
	0.00	10.03	8.70	79.97	29.88	43.48	11.19	42.14	16.94	49.16
	4.40	10.03	0.00	10.03	26.93	39.46	16.09	42.14	20.00	48.83
	0.00	10.03	0.00	10.03	28.11	45.15	18.74	39.80	15.68	61.20
	0.00	10.03			29.75	45.48	6.64	29.77	24.97	36.45

	8.86	10.03			33.79	50.84	4.34	29.77	31.80	29.10
	1.50	80.00			20.37	38.13	10.94	38.46	17.50	44.48
	0.00	10.03			26.92	46.82	8.10	43.14	27.25	28.76
	7.59	20.74			18.77	45.48	9.75	29.77	21.80	39.13
**AV**	**2.24**	**30.18**	**3.21**	**39.61**	**29.77**	**44.94**	**10.17**	**37.11**	**21.12**	**36.27**
**SD**	**3.64**	**29.78**	**3.10**	**32.79**	**10.57**	**5.13**	**5.93**	**17.45**	**8.28**	**15.98**
**N**	**35.00**	**35.00**	**19.00**	**19.00**	**32.00**	**32.00**	**31.00**	**31.00**	**27.00**	**27.00**

N: number of observations, AV: average, SD: standard deviation.

Appendix Table S15. Additional FRET efficiencies and Binding used for Fig 5, Fig 7 and Appendix Fig S3.

**Date**	**WOX5-mV**		**WOX5-mV free mCh**		**WOX5-mV PLT3-mCh**		**WOX5-mV PLT3ΔPrD-mCh**	
	FRET E	Binding	FRET E	Binding	FRET E	Binding	FRET E	Binding
**29.06.2018**	10	0.00986	60	0.55	25	14		
	66	0.98	29	5.3	27	25		
	10	0.00472	28	1.6	45	59		
	10	0.17	33	2	31	25		
	80	0.56	35	2.7	42	65		
	36	5.2	34	4.3	26	20		
	62	0.62	29	4.1	34	28		
	10	0.003	18	10	39	36		
	10	0.002	25	7	40	43		
	29	1.4	19	5	32	32		
**04.07.2018**	10	0.46	34	6.2	51	7.3		
	55	2.5	49	6.3	45	39		
	29	2.8	30	9.8	39	29		
	10	0.001	32	16	36	34.1		
	10	0.46	30	4.3	38	37		
	10.133	0.84	34	9.4	39	35		
	10.1	0.001	22	14	39	40		
	10.1	0.001	23	19	39	26		
	16	4.3	42	5.4	43	21		
	10	11	33	7.6	41	40		
**13.05.2019**	10	0.0045	23	0.35	26	13	53	3.5
	10	0.00702	10	6	28	9.5	35	1.1
	10	0.00695	22	1.2	35	23	56	2
	80	0	31	8.6	11	36	9.7	0.001
	11	6.9	34	20	9.7	4	47	13
	19	7.7	31	24	31	8.3	37	29
	80	0.5	10	1.3	23	15	40	38
	10	0.00508	37	2.9	30	23	43	53
	10	0.00562	18	7.6	35	39	38	43
	10	0.00828	15	19	35	33	31	14
					37	39	42	8.5

					35	35	39	12
							35	23
							37	15
							26	14
							29	13
							42	32
							27	6.5
							38	21
							39	26
**14.05.2019**	80	0.3	80	0.7			32	9.2
	16	7.7	15	1.6			39	18
	80	0.16	80	0.62			26	18
	9.87	0.001	35	28			35	13
	80	0.00238	18	9.8			35	26
	9.87	0.001	9.87	0.001			31	18
	80	0	9.87	1.7			34	23
	10	0.001					31	8.9
	9.87	8.5					32	11
	9.87	0.00138					38	20
	9.87	0.001					31	19
	9.87	0.6					37	40
**20.12.2019**	80	3.5			71	11		
	80	8.9			53	44		
	80	3.4			57	34		
	80	5.1			56	40		
	72	4.4			56	43		
	80	9.4			62	27		
	80	7.9			55	48		
	80	5.3			54	41		
	80	4.2			56	26		
	80	2.3			52	36		
**n**	**52**	**52**	**37**	**37**	**42**	**42**	**32**	**32**
**AV**	**37.32**	**2.27**	**30.21**	**7.40**	**39.49**	**30.55**	**35.77**	**18.49**
**SD**	**31.88**	**3.12**	**15.99**	**6.99**	**12.80**	**13.29**	**8.19**	**12.37**

N: number of observations, AV: average, SD: standard deviation.

Appendix Table S16. FRET efficiencies and Binding related to Fig 6 and Fig 7.

Date	BRAVO-mV		BRAVO-mV mCherry-NLS		BRAVO-mV PLT3-mCh		BRAVO-mV PLT3ΔPrD-mCh	
	**FRET E**	**Binding**	**FRET E**	**Binding**	**FRET E**	**Binding**	**FRET E**	**Binding**
**23.03.2021**	9.71	0.00			48	27	52	14
	9.71	0.00			51	19	49	23
	70.00	2.10			48	33	76	4.8
	76.00	3.40			58	8.2	79.9	5.6
	80.00	8.50			42	17	59	6.3
	10.00	0.00			43	7.7	79.9	4.7
	48.00	0.50			53	21	63	4.5
	9.71	0.00			45	35	43	7.9
	79.90	3.60			43	27	43	29
	9.71	0.00			56	22	43	44
**06.04.2021**	10.00	-10.00	56.00	0.81	61.00	7.50	54.00	7.10
	11.00	-3.70	46.00	1.90	78.00	3.70	65.00	2.90
	10.10	-8.00	80.00	1.00	43.00	27.00	42.00	23.00
	15.00	6.20	67.00	3.30	47.00	21	41.00	18.00
	14.00	10.00	71.00	3.70	42.00	25.00	80.00	0.50
	10.00	-1.50			48.00	24	9.70	-0.80
	33.00	4.30			48.00	19.00	64.00	2.60
	80.00	-3.60			51.00	19.00		
	80.00	0.18			54.00	20.00		
	14.00	5.00			46.00	38.00		
**21.05.2021**	80.00	7.80	62.00	2.80	50.00	29.00	46.00	12
	76.00	5.60	58.00	8.60	47.00	13.00	45.00	21.00
	45.00	0.29	35.00	7.30	52.00	32.00	45.00	9.10
	69.00	9.00	61.00	4.80	49.00	16.00	34.00	5.60
	54.00	4.30	56.00	4.90	47.00	23	35.00	8.80
	36.00	7.10			49.00	29.60	47.00	6.60
	9.93	0.00			33.00	13.00	39.00	12.00
	9.93	0.01			47.00	43.00	37.00	19.00
	9.93	0.01			47.00	50.60	40.00	14.00
	9.93	0.01			47.00	15.00	29.00	14.00
							37.00	8.90
**AV**	**35.99**	**1.70**	**59.20**	**3.91**	**49.10**	**22.84**	**49.20**	**11.72**
**SD**	**29.38**	**4.59**	**11.94**	**2.43**	**7.50**	**10.53**	**16.38**	**9.58**
**n**	**30.00**	**30.00**	**10.00**	**10.00**	**30.00**	**30.00**	**28.00**	**28.00**

N: number of observations, AV: average, SD: standard deviation.

Appendix Table S17. FRET efficiency and Binding values corresponding to Fig EV4.

Date	BES1-mV		BES1-mV mCherry-NLS		BES1-mV PLT3-mCh		BES1-mV PLT3ΔPrD-mCh	
	FRET E	Binding	FRET E	Binding	FRET E	Binding	FRET E	Binding
21.05.2021	9.91	-10.00	80.00	20.00	68.00	16.00	34.00	13.00
	10.00	-1.30	48.00	2.60	48.00	7.70	44.00	7.10
	80.00	0.95	46.00	5.70	45.00	9.90	54.00	8.00
	39.00	0.78	45.00	12.00				
	9.91	-2.40	50.00	7.60				
	54.00	3.40						
	18.00	4.40						
	9.91	-13.00						
	9.91	-12.00						
	9.91	-5.60						
27.04.2021	80.00	3.40	39.00	0.73	76.00	6.50	57.00	4.40
	80.00	0.00	60.00	5.70	59.00	8.90	54.00	6.30
	10.10	0.00	80.10	4.30	48.00	19.00	57.00	7.10
	10.10	0.00	80.00	1.00	43.00	23.00	45.00	18.00
	59.00	4.40	65.00	3.20	51.00	24.00	47.00	18.00
	67.00	4.40			45.00	35.00	13.00	2.20
	70.00	9.20			66.00	8.10	76.00	6.00
	55.00	9.70			55.00	6.10	56.00	3.50
	80.00	2.30			49.00	9.90	67.00	6.70
	80.00	2.00			46.00	22.00	51.00	8.3
28.05.2021	80.00	-3.50	10.00	-12.00	56.00	9.90		
	80.00	0.67	77.00	3.2	41.00	18.00		
	10.00	-12.00	63.00	7.00	45.00	20.00		
	80.00	7.50	49.00	7.00	47.00	26.00		
	80.00	-1.40	48.00	5.60	65.00	7.30		
	10.00	-12.00			42.00	23.00		
	80.00	1.40			42.00	27.00		
	80.00	6.60			45.00	28.00		
	57.00	7.20			48.00	19.00		
	64.00	5.10			46.00	14.00		
**AV**	**48.76**	**0.01**	**56.01**	**4.91**	**51.13**	**16.88**	**50.38**	**8.35**
**SD**	**30.59**	**6.34**	**18.55**	**6.46**	**9.34**	**8.09**	**14.74**	**4.81**
**n**	**30.00**	**30.00**	**15.00**	**15.00**	**23.00**	**23.00**	**13.00**	**13.00**

N: number of observations, AV: average, SD: standard deviation.

Appendix Table S18. FRET efficiency and Binding values corresponding to Fig EV4.

Date	TPL-mV		TPL-mV mCherry-NLS		TPL-mV PLT3-mCh		TPL-mV PLT3ΔPrD-mCh	
	**FRET E**	**Binding**	**FRET E**	**Binding**	**FRET E**	**Binding**	**FRET E**	**Binding**
12.04.2021	10.10	0.01	42.00	6.20	58.00	7.40	32.00	8.00
	10.10	0.00	53.00	3.50	40.00	16.00	35.00	7.00
	10.10	0.00	14.00	5.00	35.00	13.00	35.00	9
	10.00	0.01	47.00	6.90	36.00	8.00	41.00	7.60
	49.00	0.65	24.00	5.00	25.00	11.00	32.00	6.00
	55.00	2.20			41.00	16.00	46.00	12.00
	73.00	6.30			29.00	17.00	33.00	14.00
	10.10	0.01			30.00	9.50	50.00	9.7
	10.00	0.01			47.00	8.00	32.00	17.00
	30.00	5.10			35.00	12.00	43.00	11.00
					34.00	16.00	34.00	11.00
					25.00	19.00	36.00	16.00
					36.00	20.00	35.00	7.10
					34.00	18.00	27.00	6.80
					29.00	22.00	44.00	24.00
06.04.2021	10.00	-18.00	74.00	3.10	43.00	7.70	35.00	2.60
	10.00	-7.30	40.00	10.00	50.00	6.70	51.00	3.60
	10.00	5.10	47.00	7.70	42.00	12.00	64.00	2.90
	10.00	-0.80	39.00	6.00	46.00	9.80	43.00	11.00
	10.00	8.60	48.00	11.00	42.00	16.00	59.00	3.50
	11.00	3.40			46.00	11.00	26.00	4.60
	15.00	1.80			48.00	15.00	70.00	5.80
	10.00	-2.00			44.00	17.00	47.00	13.00
	27.00	0.86			47.00	19.00	47.00	12.00
	10.00	6.80			29.00	18.00	80.00	-0.48
**AV**	**19.52**	**0.64**	**42.80**	**6.44**	**38.84**	**13.80**	**43.08**	**8.99**
**SD**	**17.91**	**5.51**	**15.32**	**2.44**	**8.31**	**4.42**	**13.19**	**5.26**
**n**	**20.00**	**20.00**	**10.00**	**10.00**	**25.00**	**25.00**	**25.00**	**25.00**

N: number of observations, AV: average, SD: standard deviation.

Appendix Table S19. Ratio of periclinal cell divisions in the QC related to Fig 7.

Treatment	Genotype	Periclinal cell division planes in the QC [%]	Number of analysed roots
DMSO	*Col-0*	27	44
	plt3-1	73	45
	plt3-1, pWOX5:GR-PLT3-mT2	83	41
	plt3-1, pWOX5:GR-PLT3dPrD-mT2	94	36
DEX	*Col-0*	28	39
	plt3-1	87	46
	plt3-1, pWOX5:GR-PLT3-mT2	67	45
	plt3-1, pWOX5:GR-PLT3dPrD-mT2	100	36

Appendix Table S20. FRET efficiencies and Binding related to Appendix Fig S7.

**Date**	**Measurement**	**WOX5-mV(N) BRAVO-mV(C)**		**WOX5-mV(N) BRAVO-mV(C) mCherry-NLS**		**WOX5-mV(N) BRAVO-mV(C) PLT3-mCh**		**WOX5-mV(N) BRAVO-mV(C) PLT3ΔPrD-mCh**	
		**Binding [%]**	**FRET E [%]**	**Binding [%]**	**FRET E [%]**	**Binding [%]**	**FRET E [%]**	**Binding [%]**	**FRET E [%]**
**20.08.24**	#1	0.00	80.03	4.99	10.93	12.72	33.44	18.21	33.44
	#2	2.36	80.03	3.13	33.77	27.33	42.38	22.51	28.15
	#3	0.28	10.60	2.40	40.40	43.22	47.68	30.88	43.71
	#4	3.28	69.21	10.50	23.84	29.10	38.74	29.72	41.72
	#5	8.20	10.26	4.50	53.64	27.10	46.69	18.30	41.39
	#6	0.00	10.60	7.92	43.71	37.48	44.04	41.26	38.41
	#7	8.50	10.26	10.26	25.83	29.62	37.09	24.80	47.02
	#8	0.00	80.13			23.60	44.37	36.14	46.69
	#9	0.00	10.26			32.83	46.36	15.97	40.07
	#10	10.23	32.45			26.32	39.74	25.29	43.71
	#11	18.31	42.05			31.27	48.01	23.36	45.70
	#12	0.00	80.13			44.78	46.69	28.82	45.70
	#13	1.65	80.03			30.58	40.07	37.63	44.11
	#14	0.63	17.22					33.55	47.68
	#15	0.00	10.26					34.04	39.74
**23.08.2024**	#1	0.00	9.90	4.07	47.19	35.90	46.86	33.78	38.61
	#2	0.00	80.20	15.90	23.76	26.47	36.96	20.18	33.99
	#3	2.73	74.26	19.20	9.90	28.93	46.86	43.96	37.59
	#4	0.00	9.90	8.00	30.69	41.81	41.25	32.10	40.59
	#5	0.71	9.90	8.97	24.87	52.31	46.20	43.33	37.62
	#6	0.00	9.90						
	#7	0.00	80.00						
	#8	1.02	9.90						
	#9	1.27	73.93						
	#10	0.56	9.90						

	#11	1.80	14.19						
	#12	1.10	60.40						
	#13	15.81	10.00						
	#14	4.10	37.29						
	#15	7.53	51.16						
**13.09.2024**	#1	0.00	10.00	3.14	10.00	16.57	40.00	25.08	29.87
	#2	0.70	10.00	3.50	13.00	29.90	31.00	19.75	27.33
	#3	2.20	79.97	3.60	79.97	31.31	40.00	42.74	35.33
	#4	11.34	36.67	15.90	13.33	34.09	45.33	35.50	33.00
	#5	2.10	79.97	14.60	10.00	30.24	50.67	20.60	39.33
	#6	0.97	10.00					21.91	22.33
	#7	0.31	10.00					20.01	28.00
	#8	0.00	80.00						
	#9	8.00	10.00						
	#10	5.19	16.67						
**AV**		**3.02**	**37.69**	**8.27**	**29.11**	**31.46**	**42.63**	**28.87**	**38.18**
**SD**		**4.48**	**30.60**	**5.20**	**18.64**	**8.51**	**4.91**	**8.40**	**6.68**
**n**		**40.00**	**40.00**	**17.00**	**17.00**	**23.00**	**23.00**	**27.00**	**27.00**

N: number of observations, AV: average, SD: standard deviation.

Appendix Table S21. Protein level values used for the simulations of protein complex formation in the root stem cell niche, based on the measured fluorescence intensity values related to Fig 1.

Cell type	pPLT3:PLT3-mV	pWOX5:WOX5mV	pBRAVO:BRAVO-mV
**SI**	0.711044	0.524895	1.001838
**QC**	0.68113	0.764438	0.486001
**CSC**	0.790596	0.327142	0.220858
**CC**	0.294725	0.053709	0.033101

SI: stele initials; QC: quiescent center, CSC: columella stem cell; CC: columella cell; mV: mVenus.

References

Betegón-Putze I, Mercadal J, Bosch N, Planas-Riverola A, Marquès-Bueno M, Vilarrasa-Blasi J, Frigola D, Burkart RC, Martínez C, Conesa A et al (2021) Precise transcriptional control of cellular quiescence by BRAVO/WOX5 complex in Arabidopsis roots. *Mol Syst Biol* 17: e9864

Burkart RC, Strotmann VI, Kirschner GK, Akinci A, Czempik L, Dolata A, Maizel A, Weidtkamp-Peters S, Stahl Y (2022) PLETHORA-WOX5 interaction and subnuclear localization control Arabidopsis root stem cell maintenance. *EMBO Rep* 23: e54105

Denninger P, Reichelt A, Schmidt VAF, Mehlhorn DG, Asseck LY, Stanley CE, Keinath NF, Evers J-F, Grefen C, Grossmann G (2019) Distinct RopGEFs Successively Drive Polarization and Outgrowth of Root Hairs. *Curr Biol* 29: 1854-1865.e5

Galinha C, Hofhuis H, Luijten M, Willemsen V, Blilou I, Heidstra R, Scheres B (2007) PLETHORA proteins as dose-dependent master regulators of Arabidopsis root development. *Nature* 449: 1053–1057

Lampropoulos A, Sutikovic Z, Wenzl C, Maegele I, Lohmann JU, Forner J (2013) GreenGate---a novel, versatile, and efficient cloning system for plant transgenesis. *PLoS One* 8: e83043

Maika JE, Krämer B, Strotmann VI, Wellmer F, Weidtkamp-Peters S, Stahl Y, Simon R (2023) One pattern analysis (OPA) for the quantitative determination of protein interactions in plant cells. *Plant Methods* 19: 73

Vilarrasa-Blasi J, González-García M-P, Frigola D, Fàbregas N, Alexiou KG, López-Bigas N, Rivas S, Jauneau A, Lohmann JU, Benfey PN et al (2014) Regulation of plant stem cell quiescence by a brassinosteroid signaling module. *Dev Cell* 30: 36–47
