# Supplementary material for: Root stem cell homeostasis in Arabidopsis involves cell-type specific transcription factor complexes
Source: EMBO Rep. 2025 Mar 19;26(9):2323–46. doi: 10.1038/s44319-025-00422-8 (PMC12069552; doi:10.1038/s44319-025-00422-8)
Supplement: Supplementary file 3 — Expanded View Figures [file 44319_2025_422_MOESM3_ESM.pdf]

# Expanded View Figures

**Figure EV1. Elevated QC division frequencies negatively correlate to the number of CSC layers.**

(**A–H**) 2D histograms visualizing the combined results of the SCN staining in the respective genotype showing the number of CSC layers on the y axis and QC divisions on the x axis. Darker colors correspond to a higher number of roots showing the phenotype. Numbers of analyzed roots per genotype (biological replicates) are indicated in each graph and result from 3 to 5 technical replicates. (**I**) Close-up of the QC in the Col-0 WT. Scale bar: 5 µm. (**J**) Close-up view of the QC of a *bravo-2* mutant showing an additional periclinal cell division plane (white arrowhead). Scale bar: 5 µm. (**K**) Quantification of periclinal cell division planes in the different mutants. Correlations between periclinal QC divisions and genotype were tested with omnibus Pearson's Chi-squared test followed by pairwise comparisons with *P* value adjustment after Benjamini and Hochberg.

 © The Author(s)

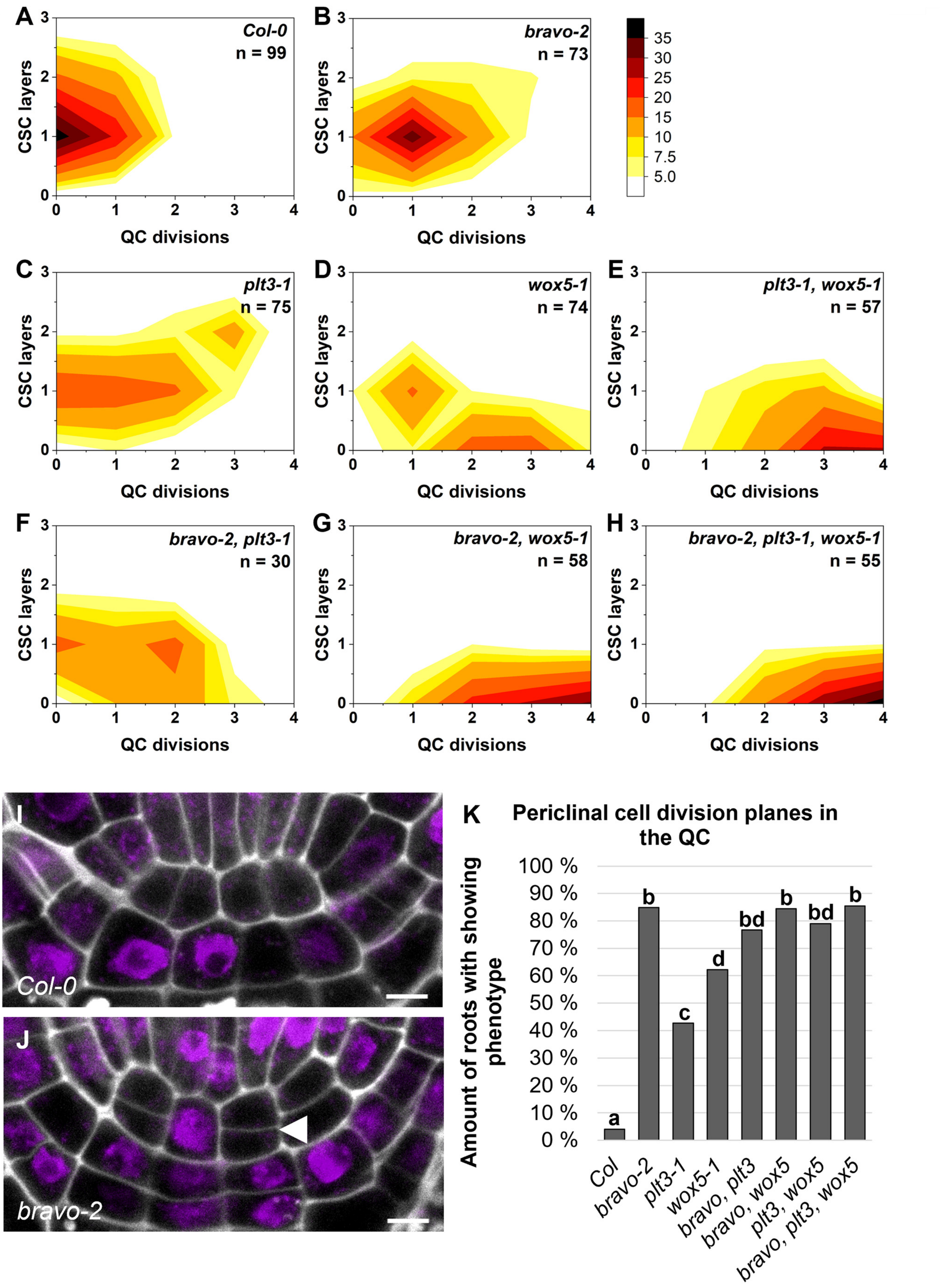
A
Col-0
n = 99
B
bravo-2
n = 73
35
30
25
20
15
10
7.5
5.0
C
plt3-1
n = 75
D
wox5-1
n = 74
E
plt3-1, wox5-1
n = 57
F
bravo-2, plt3-1
n = 30
G
bravo-2, wox5-1
n = 58
H
bravo-2, plt3-1, wox5-1
n = 55
CSC layers
QC divisions
I
Col-0
J
bravo-2
K
Periclinal cell division planes in the QC
Amount of roots with showing phenotype
100 %
90 %
80 %
70 %
60 %
50 %
40 %
30 %
20 %
10 %
0 %
a
b
c
d
bd
b
bd
b
Col
bravo-2
plt3-1
wox5-1
bravo, plt3
bravo, wox5
plt3, wox5
bravo, plt3, wox5

© The Author(s)

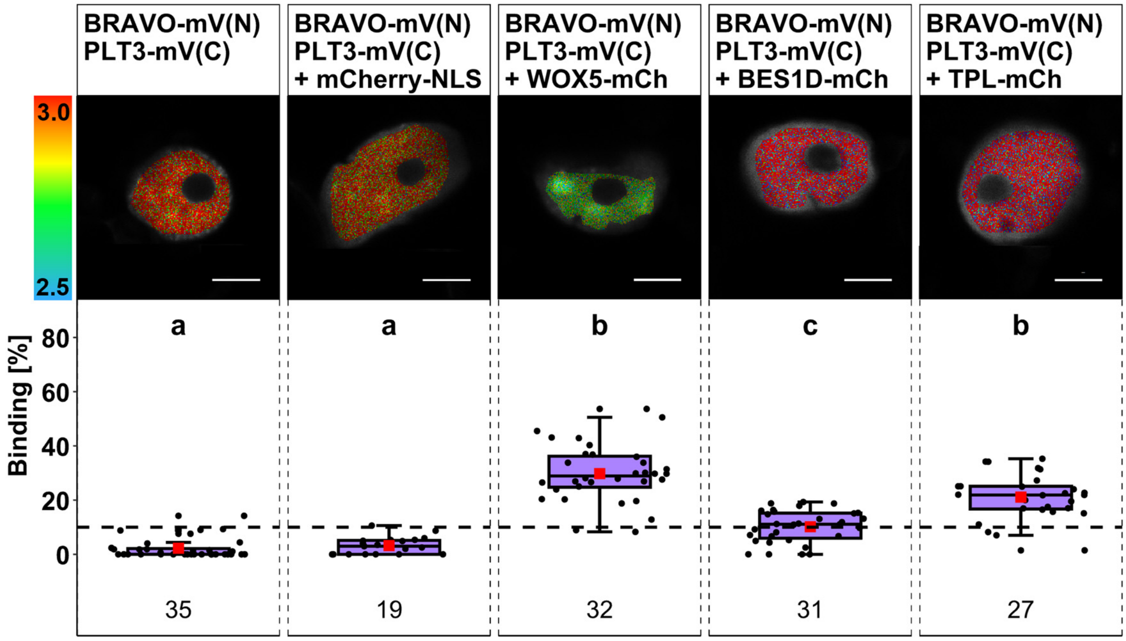


**Figure EV2. Trimeric complex formation of BRAVO and PLT3 with WOX5, BES1D and TPL.**

Upper panel: Representative images of fluorescence lifetime imaging microscopy (FLIM) measurements in *N. benthamiana* epidermal leaf cells after a pixel-wise multiexponential fit. The fluorescence lifetime of the donor BRAVO-mV(N) PLT3-mV(C) in the presence or absence of the indicated acceptor is color-coded: blue (2.5) refers to low fluorescence lifetime [in ns], red (3.0) indicates high fluorescence lifetime. Scale bars: 6 µm. Lower panel: Binding [%] (magenta) for BRAVO-mV(N) PLT3-mV(C) with or without co-expression of mCherry-NLS, WOX5-mCh, BES1D-mCh or TPL-mCh. Statistical groups were assigned after a nonparametric Kruskal–Wallis with post hoc Dunn's test (α = 0.05, *p* values were adjusted after Benjamini and Hochberg). The black dotted line indicates the Binding cutoff of 10%. The numbers of analyzed nuclei (biological replicates) are indicated below each sample and result from 3-4 technical replicates. Data Information: Box = middle 50% of data ( = interquartile range (IQR)); whiskers = from IQR to min/max values, but at most 1.5 * IQR; line within box = median; data beyond the end of whiskers are ‚outliers' and are plotted independently. In addition, a jitter plot was used to visualize all data points individually.

 © The Author(s)

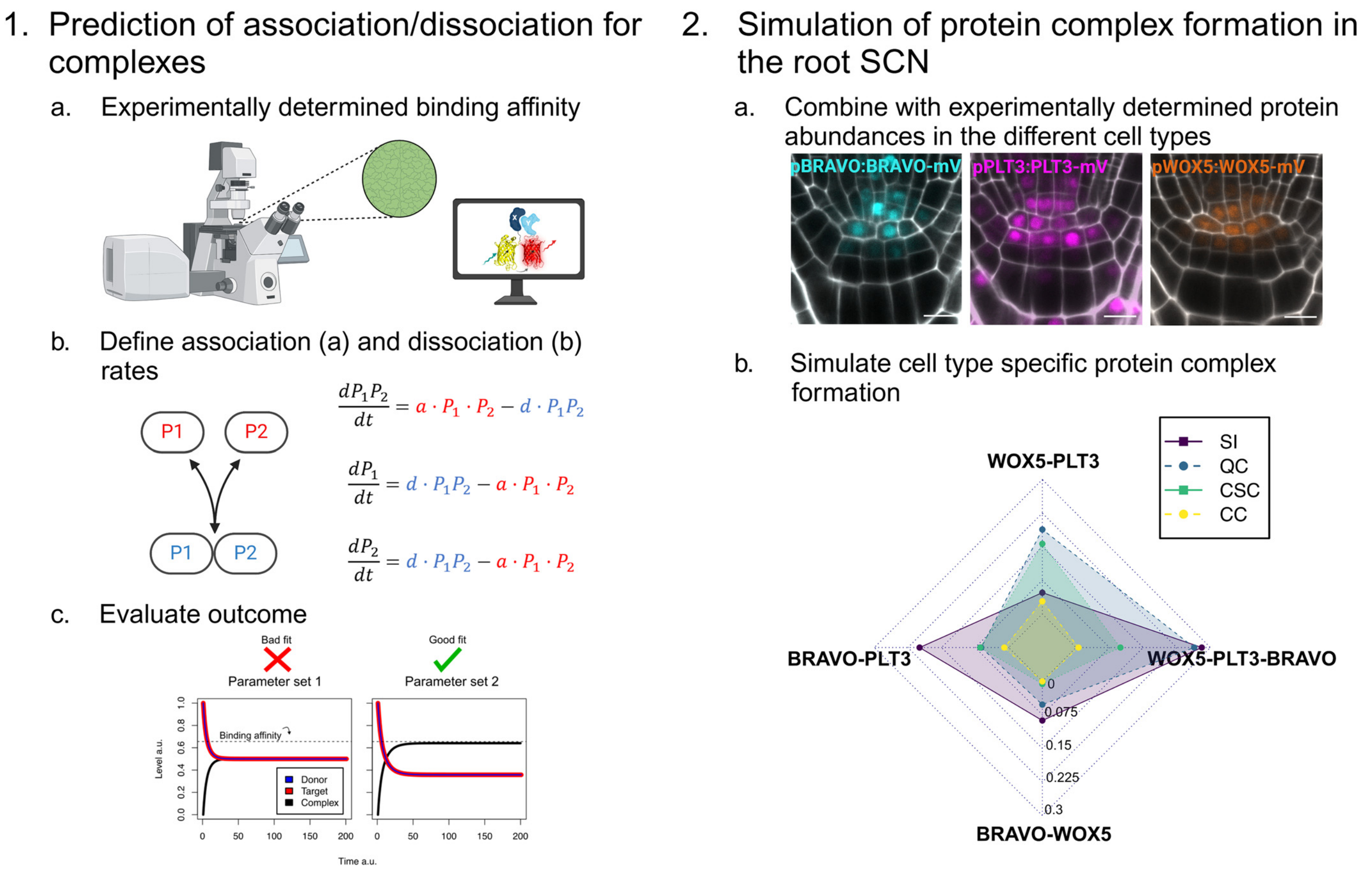


**Figure EV3. Workflow of the mathematical modeling.**

**1a**: First, we experimentally determined the binding affinity of all TF combinations. **1b**: For the simulation of protein complex formation in the root, we first defined association (a) and dissociation (d) rates for each combination of TFs and saved them as a parameter set. **1c**: Each parameter set was evaluated whether it can reproduce the experimentally determined binding affinities from our FRET-FLIM studies. If so, the parameter set was kept. For each combination, many different parameter sets were able to reproduce the experimental data. **2a**, **b:** Finally, one parameter set was randomly chosen and combined with the experimentally determined protein abundances to finally simulate the cell-type specific protein complex formation.

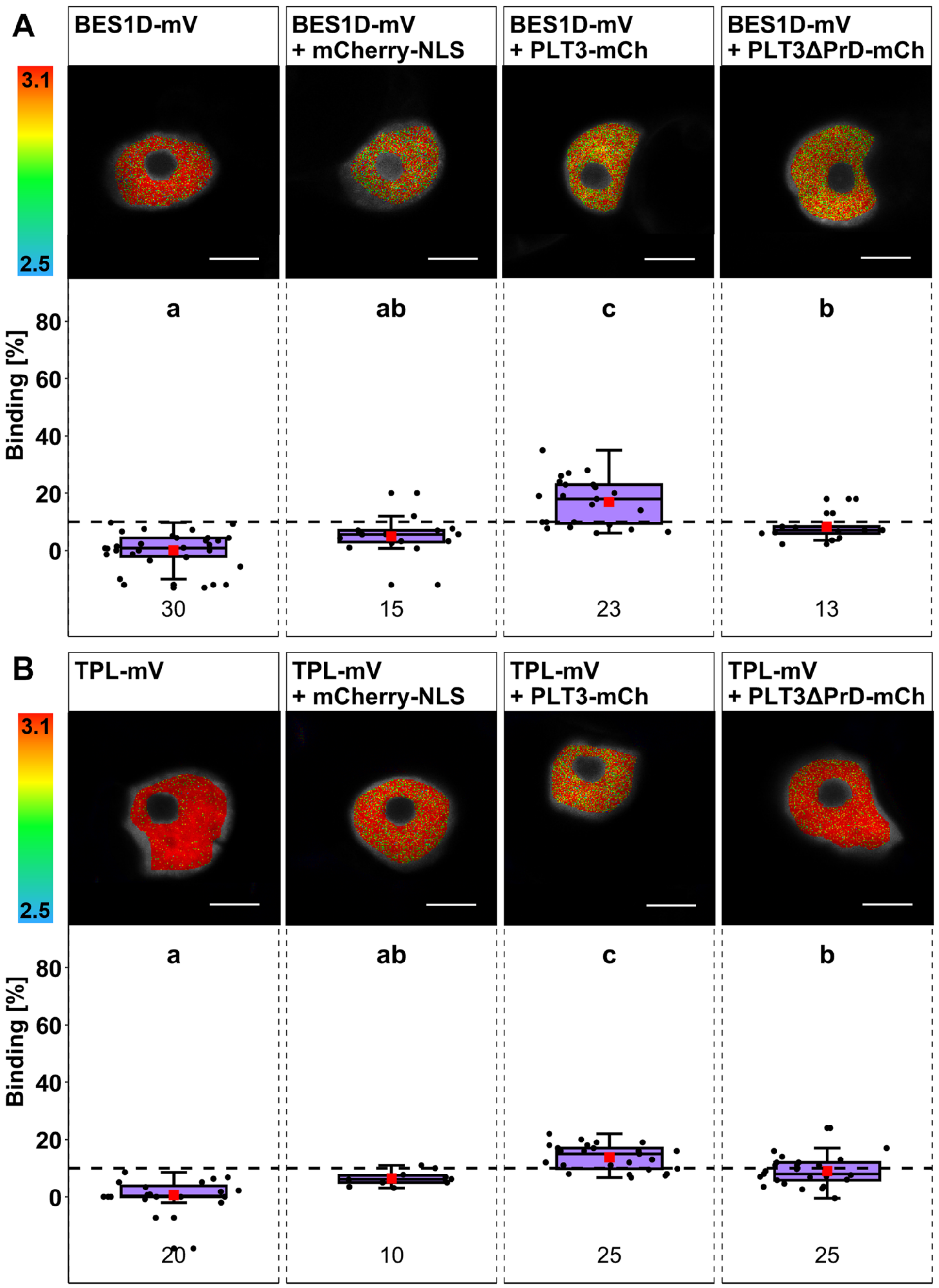


**Figure EV4. Interaction of PLT3 with BES1D and TPL depends on PrDs found in PLT3.**

(**A**) Upper panel: Representative images of fluorescence lifetime imaging microscopy (FLIM) measurements in *N. benthamiana* epidermal leaf cells after a pixel-wise multiexponential fit. The fluorescence lifetime of the donor BES1D-mV in the presence or absence of the indicated acceptor is color-coded: blue (2.5) refers to low fluorescence lifetime [in ns], red (3.1) indicates high fluorescence lifetime. Scale bar represents 6 µm. (**A**) Lower panel: Binding [%] (magenta) for BES1D-mV with or without co-expression of mCherry-NLS, PLT3-mCh or PLT3ΔPrD-mCh. Statistical groups were assigned after a nonparametric Kruskal–Wallis with post hoc Dunn's test ($\alpha = 0.05$, *p* values were adjusted after Benjamini and Hochberg). The black dotted line indicates the Binding cutoff of 10%. The numbers of analyzed nuclei (biological replicates) are indicated below each sample and result from 3 technical replicates. (**B**) Upper panel: Representative images of FLIM measurements in *N. benthamiana* epidermal leaf cells after a pixel-wise multiexponential fit. The fluorescence lifetime of the donor TPL-mV in the presence or absence of the indicated acceptor is color-coded: blue (2.5) refers to low fluorescence lifetime [in ns], red (3.1) indicates high fluorescence lifetime. Scale bar represents 6 µm. (**B**) Lower panel: Binding [%] (magenta) for TPL-mV in the absence or presence of mCherry-NLS, PLT3-mCh or PLT3ΔPrD-mCh. Statistical groups were assigned after a nonparametric Kruskal–Wallis with post hoc Dunn's test ($\alpha = 0.05$, *P* values were adjusted after Benjamini and Hochberg). The black dotted line indicates the Binding cutoff of 10%. The numbers of analyzed nuclei (biological replicates) are indicated below each sample and result from 2 technical replicates. Data information: (**A**, **B**) Box = middle 50% of data (= interquartile range (IQR)); whiskers = from IQR to min/max values, but at most 1.5 * IQR; line within box = median; data beyond the end of whiskers are "outliers" and are plotted independently. In addition, a jitter plot was used to visualize all data points individually.

 © The Author(s)
